# Traditional Herbal Medicine Discovery for the Treatment and Prevention of Pulmonary Arterial Hypertension

**DOI:** 10.3389/fphar.2021.720873

**Published:** 2021-11-09

**Authors:** Zhifeng Xue, Yixuan Li, Mengen Zhou, Zhidong Liu, Guanwei Fan, Xiaoying Wang, Yan Zhu, Jian Yang

**Affiliations:** ^1^ State Key Laboratory of Component-based Chinese Medicine, Tianjin University of Traditional Chinese Medicine, Tianjin, China; ^2^ Research and Development Center of TCM, Tianjin International Joint Academy of Biotechnology and Medicine, Tianjin, China; ^3^ Engineering Research Center of Modern Chinese Medicine Discovery and Preparation Technique, Ministry of Education, Tianjin University of Traditional Chinese Medicine, Tianjin, China; ^4^ Medical Experiment Center, First Teaching Hospital of Tianjin University of Traditional Chinese Medicine, Tianjin, China; ^5^ Tianjin Laboratory of Translational Research of TCM Prescription and Syndrome, Tianjin, China; ^6^ State Key Laboratory of Modern Chinese Medicine, Tianjin University of Traditional Chinese Medicine, Tianjin, China; ^7^ College of Traditional Chinese Medicine, Tianjin University of Traditional Chinese Medicine, Tianjin, China

**Keywords:** traditional herbal medicines, pulmonary arterial hypertension, active components, reverse pharmacology, mechanism

## Abstract

Pulmonary arterial hypertension (PAH) is characterized by pulmonary artery remodeling that may subsequently culminate in right heart failure and premature death. Although there are currently both non-pharmacological (lung transplantation, etc.) and pharmacological (Sildenafil, Bosentan, and new oral drugs on trial) therapies available, PAH remains a serious and fatal pulmonary disease. As a unique medical treatment, traditional herbal medicine (THM) treatment has gradually exerted its advantages in treating PAH worldwide through a multi-level and multi-target approach. Additionally, the potential mechanisms of THM were deciphered, including suppression of proliferation and apoptosis of pulmonary artery smooth muscle cells, controlling the processes of inflammation and oxidative stress, and regulating vasoconstriction and ion channels. In this review, the effects and mechanisms of the frequently studied compound THM, single herbal preparations, and multiple active components from THM are comprehensively summarized, as well as their related mechanisms on several classical preclinical PAH models. It is worth mentioning that sodium tanshinone IIA sulfonate sodium and tetramethylpyrazine are under clinical trials and are considered the most promoting medicines for PAH treatment. Last, reverse pharmacology, a strategy to discover THM or THM-derived components, has also been proposed here for PAH. This review discusses the current state of THM, their working mechanisms against PAH, and prospects of reverse pharmacology, which are expected to facilitate the natural anti-PAH medicine discovery and development and its bench-to-bedside transformation.

## 1 Introduction

Pulmonary arterial hypertension (PAH), usually leading to ultimate right ventricular heart failure, is a serious and fatal lung disease with high morbidity and mortality (Rosenkranz et al., 2020). The PAH prevalence is near 1% of the global population, with the proportion of people over 65 years increasing to up to 10% (Hoeper et al., 2016). Although the exact pathogenic mechanism of PAH is still poorly understood, pulmonary arterial remodeling due to the excessive proliferation of pulmonary artery smooth muscle cells (PASMCs) and the injury of pulmonary artery endothelial cells (PAECs) mainly manifested, possessing an overladen perivascular infiltration immune response involving multi-immunity cell types, such as B- and T-lymphocytes, neutrophils, and dendritic cells (Humbert et al., 2019).

With a better understanding of the molecular mechanisms underlying PAH, more treatment strategies have emerged, for example, vasodilators or antiproliferative mediators for reversing or inhibiting vasoconstriction (Zhang J. et al., 2018), the proliferation of PAECs and PASMCs, and thrombosis and mitotic pathways for ameliorating endothelial dysfunction and effectively slowing down PAH (Archer et al., 2010). The related drugs include prostacyclin (prostaglandin I2, iloprost, or treprostinil), endothelin receptor blocker (bosentan, ambrisentan, or macitentan), phosphodiesterase type 5 inhibitor (sildenafil, tadalafil, or vardenafil), or L-type calcium channel blockers. These current therapies have been shown to reduce symptoms or improve the quality of life of PAH patients. Nevertheless, the prognosis of PAH is still poor and the mortality rate is highly comparable to cancer (Xiang et al., 2018). Therefore, there is an urgent need to discover or develop more effective therapies for preventing or treating PAH.

**FIGURE 1:**
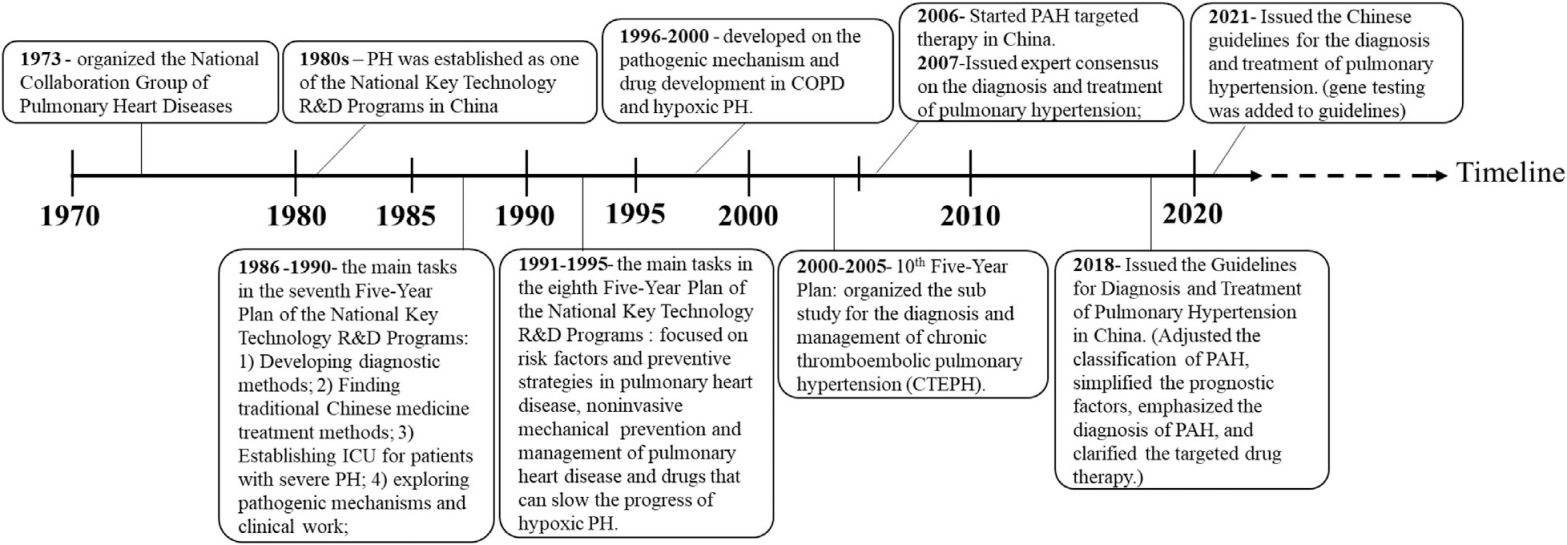
Brief history of PAH research in China.

Due to the provision of evidence-based medicine data and the development of modern medical technology in China, diagnosis and treatment approaches for PAH have improved, as well as China’s policies for PAH treatment since the 1970s, which are summarized in Figure 1. As early as 1986–1990 (the seventh 5-year plan), finding new and effective Chinese medicine treatments to improve the lung and heart function of patients with PAH has been included as the main task in the National Key Technology Research and Development Plan of China (Zhai et al., 2010). Subsequently, in 2017, a China expert consensus was issued for PAH Screening, Diagnosis, and Treatment to support clinical and scientific research. In 2018, the Chinese Guidelines for the “Diagnosis and Treatment of Pulmonary Hypertension” further adjusted the classification, simplified the prognostic factors, and emphasized the diagnosis of PAH in patients with positive pulmonary vasodilation tests and clarified the targeted drug therapy, especially focusing on the treatment of thromboembolic PAH. In the 2021 revision of “the Chinese Guidelines for the Diagnosis and Treatment of Pulmonary Hypertension,” genetic testing for PAH patients was added to prevent the occurrence and development of hereditary pulmonary hypertension (Figure 1).

Traditional herbal medicine (THM), mainly sourced from Traditional Chinese medicine (TCM), Ayurveda, and Japanese and Kampo medicine, were used to fight various diseases as early as 5,000 years ago. According to statistics, more than 80% of the population in developing countries rely on THM for basic medical care (Tang et al., 2008). As a unique medical treatment, THM treatment has gradually exerted its advantages in the treatment of PAH worldwide through a multi-level and multi-target approach. However, due to the complexity of THM components and limited analytical methods, currently, few PAH-treating THM have been approved by the FDA and CFDA. For example, among the total 1193 clinical trials reported in the U.S. National Library of Medicine (https://clinicaltrials.gov/) by March 2021, only 3 are THM-related, which include tanshinone IIA sodium sulfonate, beetroot juice and epicatechin. Meanwhile, among 60 clinical trials reported in the Chinese clinical trial registry (https:www.chictr.org.cn/index.aspx), 3 are THM-related, which are tetramethylpyrazine phosphate, rosuvastatin combined with garlic extract, and tanshinone IIA sulfonic acid sodium (Table 1).

**TABLE 1:** Current FDA and CFDA Clinical Trials for PAH involving THM components.

No	Name of Assay	Status	Identifier	Location
1*	Efficacy and safety study of sodium Tanshinone IIA sulfonate on pulmonary hypertension	Unknown	NCT01637675	The First Affiliated Hospital of Guangzhou Medical University Guangzhou, Guangdong, China
2*	(-)-Epicatechin and pulmonary arterial hypertension	Withdrawn	NCT01880866	UCSF San Francisco, California, United States
3*	BEET PAH: A study to assess the effects of beetroot juice in patients with pulmonary arterial hypertension	Completed	NCT02000856	Uppsala University Hospital Uppsala, Sweden
4	A randomized controlled pilot trial for efficacy and safety of Tetramethylpyrazine phosphate in the treatment of pulmonary hypertension	Prospective registration	ChiCTR1800018664	• The First Affiliated Hospital of Guangzhou Medical University
5	Efficacy and safety study of Tetramethylpyrazine phosphate on pulmonary hypertension: a randomized controlled pilot trial Chen et al. (2020c)	Prospective registration	ChiCTR-IPR-14005,379	• The First Affiliated Hospital of Guangzhou Medical University
6	Efficacy of rosuvastatin combined with garlic extract on patients of pulmonary hypertension: a randomized, controlled trial	Prospective registration	ChiCTR-IPR-17011827	• Union Hospital, Tongji Medical College, Huazhong University of Science and Technology
7	Efficacy and safety of Tanshinone IIA sulfonic acid sodium aerosol inhalation	Prospective registration	ChiCTR-IPR-15006669	• Guangzhou Institute of Respiratory Diseases, The First Affiliated Hospital, Guangzhou Medical University

Note: A search of keyword “pulmonary hypertension” or “PAH” in the item “Condition or disease” at https://clinicaltrials.gov/(at March 15, 2021) yielded 1193 listed studies, 3 of which were found and listed (1*-3*) in this table that are related to traditional herbs (4–7) items of the table *via* the use of search engine China clinical trial registry https: www.chictr.org.cn/index. aspx.

Although large-scale randomized controlled trials (RCT) have yet to provide convincing evidence for their therapeutic effects, clinical administration of THM, in forms such as decoctions (Tsai et al., 2008), capsules (Lu et al., 2020), granules (Kaur et al., 2015; Wang Y. et al., 2016), and injections (Jiang et al., 2016), have been widely used in daily medical care or preclinical studies. Recently, numerous preliminary studies demonstrated that THM, especially some active components derived from THM (i.e., genistein, baicalein, quercetin, and hydroxysafflor yellow A, astragalus polysaccharides, salvianolic acid A, ursolic acid, oxymatrine, berberine, tetrandrine, and ginsenoside Rb1), could serve as potential drug candidates to prevent or cure PAH (Matori et al., 2012; Zhang B. et al., 2014; Wang RX. et al., 2015; Li L. et al., 2016; Wang X. et al., 2016; Yuan LB. et al., 2017; Yuan T. et al., 2017; He et al., 2017; Hsu et al., 2018; Gao X. et al., 2020; Wande et al., 2020). These studies may help discover and develop potential safer therapies against PAH using THM.

In this review, we mainly focused on the preclinical studies of THM for treating PAH. The compound prescriptions**,** single herb, and active components from THM for PAH treatment are comprehensively summarized. In addition, the potential molecular mechanisms of THM’s therapeutic effects in classic preclinical PAH models are classified and reviewed. Last but not least, reverse pharmacology, an effective strategy for THM investigations, is proposed to guide the discovery and application of THM for treating PAH.

## 2 Preclinical Studies of Traditional Herbal Medicine for Pulmonary Arterial Hypertension Treatment

**FIGURE 2:**
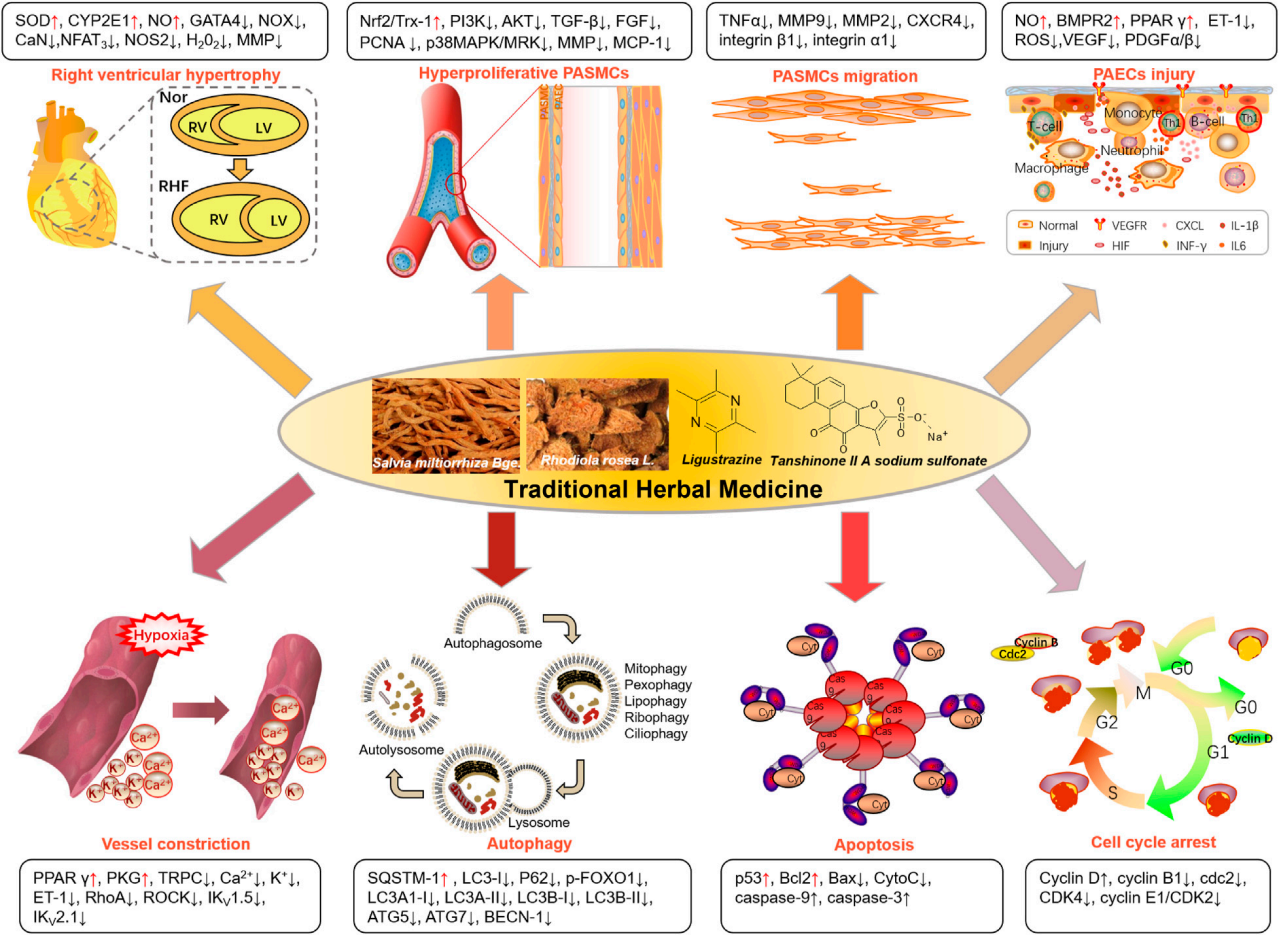
The main biological phenomenon of THM against PAH and the relative markers involved.

Increasing preclinical investigations have been reported on THM-based PAH treatment, including compound prescriptions, single herbal preparations, and molecules derived from THM. The therapeutic approaches and relative markers of the current exploration of THM for the PAH treatment were summarized in Figure 2. THM is mainly used to treat PAH by reducing right ventricular hypertrophy, inhibiting excessive proliferation and migration of PASMCs, improving pulmonary vascular remodeling caused by hypoxia, promoting apoptosis of PASMCs, and regulating the cell cycle. Taking tanshinone IIA, as an example, reduces the right heart hypertrophy index via reducing pathway (CaN-NFAT_3_) or markers (GATA4 or MMP), as detailed below.

### 2.1 Compound Traditional Herbal Medicine Prescriptions for Pulmonary Arterial Hypertension Treatment

As a natural combination medicine, multi-component THM have been shown to synergistically delay or hinder the occurrence and development of PAH. Based on the dialectical concept, THM approaches are fundamentally different from that of Western medicines in PAH diagnosis and treatment (Chan et al., 2010). The pharmacodynamics evaluation and/or diagnosis for THM treatment are more complex and comprehensive (Jiang, 2005), where qi, blood circulation, hydration, and removing phlegm are usually used as the key factors to formulate THM prescriptions. THM decoction, capsule, and injections and other forms, such as San Huang Xie Xin Tang, Qili Qiangxin capsule, and Qishen Yiqi formula, were widely applied against PAH (Tsai et al., 2008; Wang Y. et al., 2016; He et al., 2018; Lu et al., 2020; Wu et al., 2021). For instance, San Huang Xie Xin Tang, composed of *Coptidis rhizoma*, *Scutellariae radix* and *Rhei rhizoma*, significantly attenuates U46619-induced arterial blood high pressure by downregulating the expressions of phosphodiesterase type 5 (PDE5), Rho-kinase (ROCK) II, and cyclooxygenase-2 (COX-2) and upregulating the expression of soluble guanylyl cyclase 1 (sGC1) (Tsai et al., 2008). Lu et al. (2020) have reported that Qili Qiangxin capsule, composed of *Astragalus membranaceus*, *Panax ginseng* C. A. Meyer, *Aconitum carmichaelii* Debx*.*, *Salvia miltiorrhiza Bge.*, *Draba nemorosa* L., *Alisma plantago-aquatica* Linn., *Polygonatum odoratum (Mill.) Druce, Cinnamomum cassia* Presl, *Carthamus tinctorius* L., *Periploca sepium* Bge., and *Citri reticulatae pericarpium*, directly reverses RV remodeling via restoring mitochondrial structure and lessening mitochondria-dependent apoptotic pathway. In addition, network pharmacology analysis was performed to predict targets and mechanisms of Qishen Yiqi prescription for PAH treatment (Wu et al., 2021). Another study showed that the combination of *Aconiti Lateralis Radix* Praeparata and *Fritillariae Thunbergii* Bulbus significantly improves MCT-induced PAH. However, it potentially aggravated the heart injury due to the inhibition of the PDK1/Akt/PDE4D axis and subsequent synergistic activation of the βAR-Gs-PKA/CaMKII signaling pathway (Zhuang et al., 2016). However, the safety of this combination therapy was significantly improved when ginseng was applied (Huang et al., 2018).

### 2.2 Single Herbal Components for Pulmonary Arterial Hypertension Treatment

Because of the simpler formulation and curative effects, single THM has been highly recognized and widely investigated. Preparations from Chinese natural plants, such as *Allium sativum, Salvia miltiorrhiza* Bge.*, Withania somnifera,* and *Rhodiola rosea* Linn., were used against PAH (Wang T. et al., 2020; Xiang et al., 2018) (Table 2). *In vitro* evaluation confirmed their anti-inflammatory and antioxidant activities. The potential mechanisms were further clarified in *in vivo* studies, including pulmonary vasoconstriction inducement (Fallon et al., 1998; Bombicz et al., 2017), alleviating pulmonary vascular remodeling and anti-oxidative stress response.

**TABLE 2:** Single THM in the PAH treatment.

Latin binomial	Origin	Part used	Experimental animal model	Dose (mg/kg body weight)	Pharmaceutical effects	References
*Allium sativum* L.	*Allium*	Bulbous	Hypoxia-induced PAH	100	Inhibits pulmonary vasoconstriction	Fallon et al. (1998)
*Allium ursinum* L.	*Allium*	Leaf	MCT-induced PAH for 8 weeks	20,000	The beneficial effects on PAH did not depend on PED5	Bombicz et al. (2017)
*Salvia miltiorrhiza* Bge.	*Salvia Linn*	Roots	MCT-induced PAH for 3 weeks	4,600, 14,000	Decreases ET-1 and thromboxane A2, increases NO and prostacyclin, and reduces the level of TGF-β1	Wang et al. (2015c)
*Crataegus oxyacantha*	*Crataegus* L.	Fruit	Hypobaric hypoxia stimulated to high altitude	0.05, 0.10	Increases the NO concentrations and the serum antioxidant capacities	Ahmadipour et al. (2017)
*Mimosa pigra*	*Mimosa* Linn	Leaves	Hypoxia-induced PAH for 21 days	400	Restores the endothelium function and increases the endothelial NO synthase	Rakotomalala et al. (2013)
*Panax notoginseng*	*Araliaceae*	Roots and rhizomes	Hypoxia-induced PAH for 4 weeks	50	Decreases p38MAPK level and increases the NO level	Zhao et al. (2015)
*Allium macrostemon* Bunge	*Allium*	Bulbs	PE-contracted PA rings	100–1000	Induces relaxation in PAs via an endothelium-dependent mechanism involving Ca^2+^ entry, PK-dependent NOS phosphorylation, and NO signaling	Han et al. (2017)
*Ocimum sanctum*	*Ocimum*	Leaves	MCT-induced PAH for 4 weeks	200	Decreases Nox-1 expression and increases expression of Bcl2/Bax ratio	Meghwani et al. (2018)
Roxb. ex DC*.*	*Combretaceae*	Stem bark	MCT-induced PAH for 25 days	125, 250	Decreased expression of NOX1 and increases the expression of Bcl2/Bax ratio	Meghwani et al. (2017)
*Rhodiola algida*	*Rhodiola* L.	Roots	Hypoxia-induced PAH	62.5, 125, 250	Suppresses the level of PCNA, cyclin D1, and CDK4 and increases p27^Kip1^ expression	Kosanovic et al. (2013), Nan et al. (2018)
*Withania somnifera*	*Withania*	roots	MCT-induced PAH for 3 weeks	50, 100	Decreases the level of ROS, IL-10, TNFα, NFκB and HIF1α and increases the procaspase-3	Kaur et al. (2015)
*Eulophia macrobulbon*	*Eulophia*	tubers	MCT-induced PAH	15, 450, 1000	Reduces the vascular contractions and inhibits intracellular Ca^2+^ release	Wisutthathum et al. (2018)

NO and ET-1 are the key vasodilators in the processes of pulmonary vasoconstriction. Previous studies showed that *Salvia miltiorrhiza* Bge. (Wang Y. et al., 2015), *Crataegus oxyacantha* (Ahmadipour et al., 2017), *Mimosa pigra* (Rakotomalala et al., 2013), *Panax notoginseng* (Zhao et al., 2015), and *Allium macrostemon* Bunge (Han et al., 2017) increased NO production.

Pulmonary vascular remodeling is mainly manifested by the damage of PAECs and apoptosis resistance of PASMCs. After treating with *Withania somnifera,* the levels of NFκB and HIF1α were decreased, and procaspase-3 increased significantly. Therapies using *Ocimum sanctum* (Meghwani et al., 2018) or Roxb. ex DC*.* (Meghwani et al., 2017) decreased the ratio of apoptosis marker proteins; Bcl2/Bax. *Rhodiola algida* was demonstrated to significantly improve pulmonary vascular remodeling due to its multi-functions, i.e.*,* decreasing expression levels of PCNA, cyclin D1, and CDK4, inhibiting degradation of p27^Kip1^, and improving chronic pulmonary vasoconstriction, vasoproliferation, and vascular inflammation (Kosanovic et al., 2013; Nan et al., 2018).

In addition, the regulation of Ca^2+^ is one of the effects of *Salvia miltiorrhiza* Bge. on PAH (Wang Y. et al., 2015)*.* Moreover, *Panax notoginseng* reduces the expression of p38MAPK that has been demonstrated to be an underlying mechanism in the generation of PAH (Zhao et al., 2015).

### 2.3 Active Components From Traditional Herbal Medicine for Pulmonary Arterial Hypertension Treatment

There is accumulating evidence for the potential benefits of active THM components in the treatment of PAH. They were reported with clear pharmacodynamic and pharmacokinetic efficacy and working mechanisms. These components under preclinical studies are classified into the following categories: flavonoids, alkaloids, glycosides, phenolic acids, polysaccharides, terpenes, and volatile oil compounds.

#### 2.3.1 Traditional Herbal Medicine-Derived Flavonoids

**FIGURE 3:**
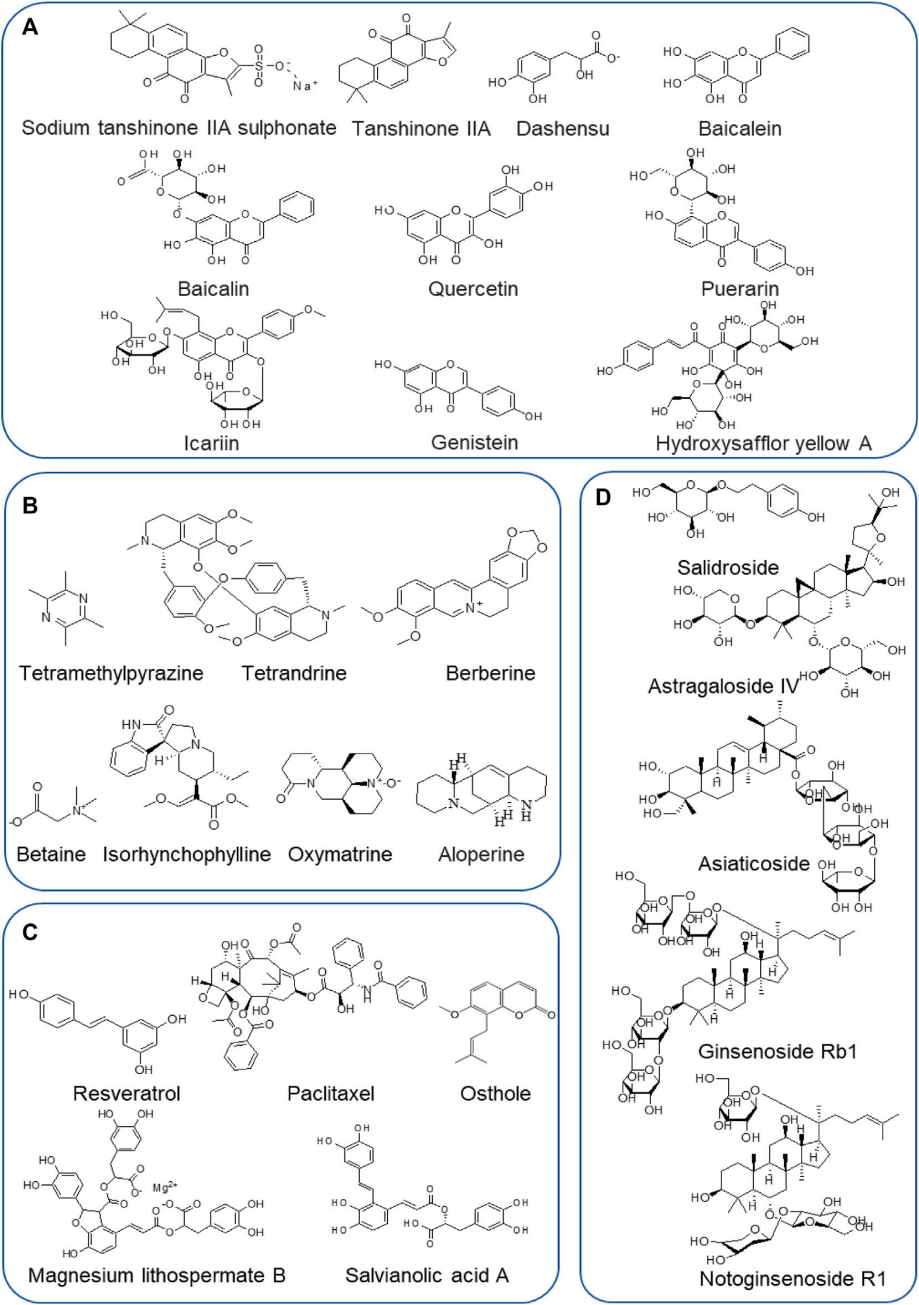
Chemical structures of frequently studied THM-derived active components against PAH. **(A)** Flavonoids. **(B)** Alkaloids. **(C)** Phenolic acids. **(D)** Glycosides.

Flavonoids are a ubiquitous group of natural compounds characterized by the flavan nucleus, possessing antibacterial, antiviral, and anti-inflammatory effects. Table 3 shows ten frequently studied flavonoids (sodium tanshinone IIA sulphonate, tanshinone IIA, dashensu, quercetin, baicalin, baicalein, puerarin, icariin, genistein, and hydroxysafflor yellow A) derived from THM that have been reported with anti-PAH activities. The chemical structures of these flavonoids are depicted in Figure 3A.

**TABLE 3:** Flavonoids as THM-derived active components for PAH treatment.

Active components	Experimental model	Dose (mg/kg body weight)	Cellular targets	Mechanisms identified	References
Sodium tanshinone IIA sulphonate	Chronic hypoxia-induced PAH	• 30	PASMCs	• PKG↑, PPAR-γ↑, TRPC1↓, TRPC6↓, SOCE↓	(Huang et al., 2009; Wang et al., 2013; Jiang et al., 2016; Bao et al., 2020)
		• 10, 30	PASMCs	• Bax/Bcl2↑; PI3K↓, *p*-Akt↓, mTOR↓, p- mTOR mTOR↓; S6K1↓, p-S6K1↓, LC3-I↑, LC3-II↑, Beclin-1↑, p62↓; IL-6↓, IL-8↓, TNF-α↓	
		• 10	PASMCs	• Kv2.1↓	
Tanshinone IIA	MCT-induced PAH	• 10	—	• TRPC1↓, TRPC6↓, [Ca^2+^]_i_↓, SOCE↓	Wang et al. (2013), Wang et al. (2010), Luo et al. (2013), Zheng et al. (2015)
	Hypoxia-induced PA	—	PAs	• Extracellular Ca^2+^ influx↓, intracellular Ca^2+^ release↓; Ca^2+^-activated K^+^ channels↑	
	Hypoxic PASMCs	—	PASMCs	• Arrests in G1/G0-phase; *p*-Akt↓/Skp2↓, p27↑	
	Hypoxia-induced PAH	• 10	PASMCs	• Kv2.1↑, Kv1.5↑, *I*_Kv_ currents↑	
Dashensu	Hypoxia-induced PAH	• 80, 160, 320	PASMCs	• TGF-β↓, *p*-Smad3↓	Zhang et al. (2018c), Liu et al. (2020a)
Quercetin	Hypoxia-induced PAH	• 100	PASMCs	• FOXO1↑, SENS3↑, *p*-mTOR/mTOR↓, p-P70 S6K/P70 S6K↓, p-4E-BPI/4E-BPI↓, LC3-I↑, LC3-II↑, p62↓; cleaved-PARP↑, cleaved-caspase3↑, cleaved-caspase9↑	He et al. (2015), He et al. (2017), Cao et al. (2019b)
		• 100	PASMCs	• p-TrKA/TrKA↑, *p*-Akt/Akt↓, arrest G1/G0-phase, cyclin D1↑, cyclin B1↓, Cdc2↓; Bax/Bcl-2↑; MMP2↓, MMP9↓, CXCR4↓, integrin β1↓, integrin α5↓	
	Hypoxic PASMCs	—	PASMCs	• GRP78↑, p-IRE1↑, ATF6↑, *p*-eIF2a↓	
	MCT-induced PAH	• 100	—	• PCNA↓	Gao et al. (2012), Rajabi et al. (2020)
		• 30	—	• miR-204↑, PARP1↓, HIF1α↓, NFATc2↓, α-SMA↓, IL-1β↓, IL-8↓	
Baicalin	Hypoxia-induced PAH	• 30	—	• ADAMTS-1↑, collagen I↓	Zhang et al. (2014b), Huang et al. (2014), Liu et al. (2015), Huang et al. (2017)
		• 100	PASMCs	• p-Akt/Akt↓, HIF-α↓, p27↑	
		• 60	—	• A_2A_R↑, SDF-1↓, CXCR4↓, p-PI3K/PI3K↓, *p*-Akt/Akt↓	
	Hypoxic PASMCs MCT-induced PAH Hypoxic PASMCs	—	PASMCs	• HIF1α↓, AhR↓	
		• 100	PASMCs	• TNF-α↓, IL-1β↓, IL-6↓, p-NF-κB/NF-κB↓, VCAM↓, ICAM↓, BMPR2↑, p- Smad 1/5/8↑, ID1↑; Cycclin B1↓, p27↑	Luan et al. (2015), Zhang et al. (2017b), Xue et al. (2021)
		• 100	—	• NF-κB p65↓, IκB↑, BMP2↑, BMPR2↑, BMP4↑, BMP9↑, ID1↑, ID3↑, Smad1/5/8↑; gremlin-1↓, TGF-β1↓, *p*-Smad2/3↓; CD31↑, E-cadherin↑, α-SMA↓	
		• 100	—	• ET-1↓	
Baicalein	MCT-induced PAH Hypoxic PASMCs	• 100	PASMCs	• IL-6↓, TNF-α↓, IL-1β↓; MDA↓, SOD↑, GSH-Px↑; PCNA↓, p-p38/p38↓, *p*-ERK/ERK↓, *p*-JNK/JNK↓, NF-κB↓	Shi et al. (2018a), Shi et al. (2018b), Hsu et al. (2018)
		• 50, 100	—	• p-Akt↓, *p*-ERK1/2↓, *p*-GSK3β↓, β-catenin↓, ET-1↓, ET_A_R↓; eNOS↑, iNOS↓, vWF↓	
		• 50, 100	—	• EndoMT↓ (CD31↑, N-cadherin↓, VE-cadherin↑, vimentin↓, Snail↓, Slug↓, NF-κB↓, BMPR2↑, collagen I↓, collagen III↓, LOX↓	
Puerarin	Hypoxic PASMCs	—	PASMCs	• Cytosolic cyto C↑, mitochondria cyto C↓, Bax↑, Bcl-2↓	Chen et al. (2012), Zhang et al. (2019c), Yuan et al. (2019)
	Hypoxia-induced PAH	• 80	PASMCs	• PCNA↓, Cyclin A↓, Cyclin D1↓, Cyclin E↓; LC3B-I↓, LC3B-II↓, SQSTM1↑, BECN-1↓, ATG5↓	
	Hypoxic PAECs	—	PAECs	• Bax/Bcl-2↓, NO↑, ET-1↓, ROS↓, BMPR2↑, *p*-Smad1/5↑, PPARγ↑, PI3K↑, *p*-Akt/Akt↑, *p*-eNOS↑	
Icariin	MCT-induced PAH	• 20, 40, 80	—	• eNOS↑, PDE5↓, NO↑, cGMP↑	Li et al. (2016b), Lan et al. (2018), Xiang et al. (2020)
		• 40	—	• ET-1↓	
		• 50, 100	—	• TGF-β1↓, *p*-Smad2↓, *p*-Smad3↓, MMP2↓	
Genistein	Hypoxia-induced PAH	• 60	HUVECs	• p-eNOS↑, *p*-Akt/Akt↑, EPO↑, EPOR↑	Kuriyama et al. (2014)
	MCT-induced PAH associated RHF	• 1	PASMCs	• ERβ↑	Matori et al. (2012)
Hydroxysafflor yellow A	Hypoxia-induced PAH	• 25, 50, 75, 100	PASMCs	• PCNA↓, Ki67↓	Li et al. (2016a)
	MCT-induced PAH	• 10	—	• IL-6↓, TNF-α↓, IL-1β↓; MDA↓, 8-OHdG↓, SOD↓	Han et al. (2016)

Sodium tanshinone IIA sulfonate, a water-soluble salt solution of sulfonated tanshinone IIA, is a widely used active agent extracted from *Salvia miltiorrhiza* Bge. Over the past decades, numerous publications have reported its efficacy in treating cardiovascular diseases, and importantly, with no significant side effects being reported (Zhou et al., 2019). Recent studies demonstrated that it has positive effects on the treatment of chronic hypoxia-induced PAH, predominantly by inhibiting the intracellular calcium homeostasis and proliferation of PASMCs and activating the hypoxia-inhibited PKG-PPAR-γ signaling pathway in PASMCs (Jiang et al., 2016). Sodium tanshinone IIA sulfonate also inhibits the PI3K/AKT/mTOR pathway and inflammatory responses but increases apoptosis and autophagy in hypoxia-induced PAH rats (Bao et al., 2020). Wang et al. reported that sodium tanshinone IIA could prevent PAH development primarily by suppressing TRPC1 and TRPC6 expression, resulting in restored basal [Ca^2+^]_i_ and weakened the proliferation and migration of PASMCs (Wang et al., 2013). Moreover, the inhibition of Kv2.1 channel expression was also one of the mechanisms (Huang et al., 2009). With these data, the translational effect of sodium tanshinone IIA sulfonate in treating PAH has been accomplished in China (ChiCTR-IPR-15006669).

Tanshinone IIA is different from sodium tanshinone IIA sulfonate in terms of solubility, as it is poorly soluble in water. Nevertheless, significantly positive effects on PAH were reported in previous studies, especially the vasorelaxant effect (Jin et al., 2008). The results revealed that the vasorelaxant effects were exerted *via* suppression of extracellular Ca^2+^ influx, activation of K^+^ channels in PASMCs (Wang et al., 2010; Zheng et al., 2015), and inhibition of p27 degradation via Akt/Skp2-associated pathway (Luo et al., 2013).

To sum up, there is no significant correlation between sodium tanshinone IIA sulfonate and tanshinone IIA due to their different chemical structures and pharmacokinetics and pharmacological activities, although they have very similar pharmacological effects on cardiovascular diseases or pulmonary diseases. Further clinical studies are needed to validate their efficacies and potential side effects.

Danshensu, another major active component in *Salvia miltiorrhiza*, also has various therapeutic effects on cardiovascular diseases (e.g., hypertension, atherosclerosis, myocardial ischemia, and reperfusion) (Zhang J. et al., 2019), cerebral disorders (Bae et al., 2019; Han et al., 2019), and organ fibrosis (Cao G. et al., 2019). It could prevent PAH *via* TGF-β/smad3 associated pathway in the hypoxia-induced PAH model and inhibit the proliferation of PASMCs (Zhang N. et al., 2018; Liu G. et al., 2020).

Quercetin, a well-known natural flavonoid from daily diet and herb medicines, has been frequently applied to treat a variety of diseases such as cardiovascular diseases, cancers, and metabolic diseases (Chen W. et al., 2020; Ezzati et al., 2020; Ferenczyova et al., 2020). Increasing evidence confirms that quercetin also has therapeutic effects on hypoxia or MCT-induced PAH. Mechanically, quercetin inhibited the proliferation of PASMCs via multi-signaling pathways, including the FOXO1-SENS3g-mTOR apoptosis pathway (He et al., 2017), TrkA/Akt signaling pathway (He et al., 2015), and excessive ERS and the IRE1α pathway (Cao X. et al., 2019) in hypoxia-induced PAH. In addition, quercetin improved the vascular remodeling and proliferation in MCT-induced PAH *via* regulating the expression of PARP1, miR-204, and their downstream targets, HIF1α and NFATc2 (Gao et al., 2012; Rajabi et al., 2020). A recent report by Soodeh Rajabi et al. further revealed that quercetin ameliorated right ventricular disorders in rats with PAH by decreasing inflammation, apoptosis, and fibrosis and increasing the expression of miR-204 and the antioxidant-to-oxidant ratio (Rajabi et al., 2021).

Baicalin, a monomeric flavonoid compound, is the main medicinal component of *Scutellaria baicalensis* Georgi, which has highlighted extensive pharmacological properties such as cardiovascular diseases, cancer, hypertension, ischemic heart disease, and atherosclerosis in numerous studies (Li Y. et al., 2020; Liu X. et al., 2020; Xin et al., 2020; Singh et al., 2021). In addition, a large body of publications have reported that baicalin possessed a protective effect on hypoxia-induced PAH, which was mediated by increasing the expression of ADAMTS-1 (Liu et al., 2015), targeting the Akt/HIF1α/p27-associated pathway (Zhang L. et al., 2014), enhancing A_2A_R activity, downregulating SDF-1/CXCR4-induced PI3K/Akt signaling (Huang et al., 2017), and suppressing the HIF1α and AhR pathways (Huang et al., 2014). In the MCT-induced PAH model, treatment with baicalin remarkably attenuates the pathogenesis, which was mainly associated with blocking the NF-κB signaling pathway to further trigger the BMP signaling pathway (Luan et al., 2015; Zhang Z. et al., 2017) and regulating the TNF-α signaling pathway (Xue et al., 2021). Thus, baicalin is a potential drug candidate for PAH, although more clinical research is needed in the future.

Similar to baicalin, baicalein, another major flavonoid from the roots of *Scutellaria baicalensis* Georgi, exerts several beneficial pharmacological effects such as anti-inflammatory and anti-oxidant activities and thus was used for various diseases including cancers, vascular diseases, and organ injury (Nik Salleh et al., 2020; Tuli et al., 2020). Numerous reports were focused on baicalein’s effects in treating pulmonary diseases, especially PAH, and its improvement of vascular remodeling in MCT-induced PAH was mainly by inhibiting the activation of MAPK and NF-κB pathways (Shi et al., 2018a), suppressing the Akt/Erk1/2/GSK3β/β-catenin/ET-1/ETAR signaling pathway (Hsu et al., 2018), and preventing endothelial dysfunction *via* inhibition of endothelial-to-mesenchymal transition (Shi et al., 2018b; Hsu et al., 2018).

Puerarin, usually isolated from the Chinese medicinal herb kudzu, has a positive therapeutic effect on PAH in preclinical hypoxia-induced rat models (Wang S. et al., 2020). The potent therapeutic effects are associated with enhancement of apoptosis, reduction of autophagy, and inhibition of oxidative stress (Chen et al., 2012; Zhang X. et al., 2019; Yuan et al., 2019). Sun and collagens have demonstrated that puerarin promoted apoptosis in hypoxia-induced HPASMCs *via* mitochondria-dependent pathway (Chen et al., 2012). Additionally, autophagy was tested with mRFP-GFP-LC3 in hypoxia-induced PAH and results suggested that the biomarkers of autophagy were inhibited significantly (Zhang X. et al., 2019). Furthermore, inhibition of oxidative stress and activation of the BMPR2/Smad and PPARγ/PI3K/Akt signaling pathways were also the key mechanisms for the PAH treatment by puerarin (Yuan et al., 2019).

Icariin, isolated from *Epimedium pubescens*, is a key active flavonoid of *Herba Epimedii,* which potentially protects against PAH. Increasing studies have focused the mechanisms of icariin against MCT-induced PAH and the results have shown that the improvement of vascular remodeling and inhibition of PASMCs proliferation are mainly through inhibiting the TGFβ1-Smad2/3-mediated inflammatory signaling pathway (Xiang et al., 2020) and suppressing oxidative stress that targets the NO/cGMP signaling pathway (Li LS. et al., 2016; Lan et al., 2018).

Genistein, a natural soybean-derived phytoestrogen, has been shown to have cardioprotective, vasodilator, and anti-inflammatory effects (Thangavel et al., 2019). Previous studies suggested that it could be a potential candidate drug for PAH based on its pharmacological properties. Since PAH is one of the main causes of dysfunction and failure of the right heart (Tello et al., 2021), genistein could reverse preexisting established PAH and prevent the associated RHF by restoring downregulation of estrogen receptor-β (ER-β) expression in the right ventricle and lung (Matori et al., 2012). By systematic research, Chen et al. (2019) have predicted the mechanism of action for genistein concerning PAH and found its anti-PAH effect may be closely related to PPARγ, apoptotic signaling pathway, and the nitric oxide synthesis process, which were verified by Kuriyama et al. in hypoxia-induced PAH. Moreover, they conclude that genistein has positive effects for preventing hypoxic PAH through improving PI3K/Akt-dependent, NO-mediated signaling in association with enhancement of the EPO/EPOR system (Kuriyama et al., 2014).

Hydroxysafflor yellow A, isolated from *Carthamus tinctorius* L.*,* is a key active component with powerful pharmacological activities such as antioxidant, anti-inflammatory, anticoagulant, and anticancer effects (Zhao et al., 2020). Some studies demonstrated its efficacy for treating MCT-induced PAH based on anti-inflammatory and anti-oxidant effects (Han et al., 2016). The hydroxysafflor yellow A also protected against hypoxia-induced PAH by inhibiting the proliferation of PASMCs (Li L. et al., 2016).

The epidemiological studies indicated that the progress of PAH was prevented with various sourced flavonoids such as curcumin (Rice et al., 2016), isoliquiritigenin (Jin et al., 2019), isorhamnetin (Chang et al., 2020), naringenin (Ahmed et al., 2014), rulin (Li et al., 2014), chrysin (Li XW. et al., 2015), silibinin (Zhang T. et al., 2020), formononetin (Cai et al., 2019), apigenin (He et al., 2020), and isoquercitrin (Zhang Y. et al., 2017). When treated with the flavonoid of curcumin, isoliquiritigenin, isorhamnetin, or naringenin, hypoxia- or MCT-induced PAH rodents were significantly protected, with changes in inhibited inflammatory markers such as TNF-α, IL-1β, IL-6 (Ahmed et al., 2014; Rice et al., 2016; Jin et al., 2019; Zhang T. et al., 2020; Chang et al., 2020), and BMP signaling (Chang et al., 2020). Interestingly, the oxidative stress levels in PAH rodents, including decreased ROS and elevation of NOX4, were alleviated by treatment of naringenin, rulin, and chrysin (Ahmed et al., 2014; Li et al., 2014; Li XW. et al., 2015). Regulation of apoptosis signaling pathway, as one of the pathogenic mechanisms of PAH, was investigated in some of the flavonoid studies. For example, formononetin significantly improved hypoxia-induced apoptosis in PASMCs, potentially by suppressing the PI3K/AKT and ERK pathways (Cai et al., 2019), and the other study verified that PAH was prevented by apigenin *via* inhibiting the HIF1α-Kv1.5 channel pathway to induce mitochondria-dependent apoptosis in PASMCs (He et al., 2020). In addition, isoquercitrin ameliorated MCT-induced pulmonary vascular remodeling *via* inhibiting PASMCs proliferation and blocking the PDGF-Rβ signaling pathway (Zhang Y. et al., 2017).

In summary, as the largest class of polyphenols, flavonoids have shown both chemopreventive and chemotherapeutic potential in the treatment of PAH. Further basic research and clinical study are needed to explore and validate these preclinical findings with full consideration of the side effects of flavonoids for PAH patients.

#### 2.3.2 Traditional Herbal Medicine-Derived Alkaloids

Alkaloids, with a variety of biological activities, are nitrogen-containing basic compounds of plant origins, such as Polygonaceae, Leguminosae, Apocynaceae, Solanaceae, Fangke, Rutaceae, Papaveraceaen, and Ranunculaceae. More than 3,000 distinct kinds of alkaloids have been identified. Increasing studies have focused on pharmacological activities such as anti-cancer, antiangiogenic, anti-inflammatory, and anti-proliferation (Alasvand et al., 2019; Liu C. et al., 2019; Mondal et al., 2019). In recent years, more and more researchers have also paid attention to the treatment of PAH by alkaloids. Mechanically, certain alkaloids have been shown capable of preventing and treating PAH *via* anti-inflammatory, antioxidant, autophagy, promoting PASMCs apoptosis, disrupting cell cycle, and inhibiting proliferation and migration (Guo et al., 2014; Wang X. et al., 2016; Luo et al., 2018; Chen M. et al., 2019). Therefore, the efficacies for PAH of seven frequently studied and applied alkaloids derived from THM, including tetramethylpyrazine, tetrandrine, berberine, betaine, isorhynchophylline, oxymatrine, and aloperine, along with the corresponding experimental models and identified mechanisms, are summarized in Table 4 **.** The chemical structures of these alkaloids are depicted in Figure 3B.

**TABLE 4:** Alkaloids as THM-derived active components for PAH treatment.

Active components	Experimental model	Dose (mg/kg body weight)	Cellular targets	Mechanisms identified	References
Tetramethylpyrazine	Chronic hypoxia-PAH	• 100	PASMCs	• Intracellular calcium homeostasis↓	Zhang et al. (2011), Chen et al. (2020c), Huang et al. (2021)
	Sugen/hypoxia-PAH MCT-induced PAH	• 120	PMVECSs	• ROS↓, HIF1α↓, VEGF↓	
	Hypoxia-induced vascular leakage	• 40, 80,160	PASMCs	• Arrests G0/G1-phase; p-PI3K/PI3K↓, *p*-Akt/Akt↓	
	Dogs with acute pulmonary alveolar hypoxia	• 80	—	• ET-1	Cao et al. (1998)
Tetrandrine	MCT-induced PAH	• 50	PASMCs	• Protein kinase type 1↑, iNOS↓, SOD↑, GSH↑, CAT↑, MDA↓	Wang et al. (2016a)
	Contracted PA rings and tracheal segments	• 30 μM	PAs	• Inhibits contractile responses	Wang et al. (2002)
Berberine	Sugen/hypoxia-PAH	• 20	PASMCs	• p-PP2Ac/t-PP2Ac↓, *p*-Akt/Akt↓, *p*-ERK1/2↓, p-P38↓, PCNA↓	Luo et al. (2018)
		• 20, 100	PASMCs	• BMPR-II↑, P-Smad1/5↑, TGF-β↓, *p*-Smad2/3↓, PPARγ↑	Chen et al. (2019a)
		• 100	PASMCs	• Trx1↓, β-catenin↓	Wande et al. (2020)
Betaine	MCT-induced PAH	• 100, 200, 400	—	• MCP-1↓, ET-1↓, NF-κB↓, TNF-α↓, IL-1β↓	Yang et al. (2018)
Isorhynchophylline	MCT-induced PAH	• 1000	PASMCs	• Cyclin D1↓, CDK6↓, P27Kip1↑, *p*-PDGF-Rβ↓, *p*-ERK1/2↓, p-STAT3↓	Guo et al. (2014)
Oxymatrine	Hypoxia- and monocrotaline-induced PAH	• 50	—	• MCP-1↓, IL-6↓, SDF-1↓, VCAM-1↓, ICAM-1↓, TGF-β↓, ET-1↓, VEGF↓, Nrf2↑, SOD1↑, HO-1↑; HIF1α↓, NF-κB↓; Nrf2↑, SOD↑, HO-1↑	Zhang et al. (2014a)
Aloperine	PDGF-BB-induced PASMCs proliferation MCT-induced PAH	• 25, 50, 100	PASMCs	• Arrests G0/G1-phase; NF-κB↓	Chang et al. (2019)
		• 25, 50, 100	—	• Rho A↓, ROCK1↓, ROCK2↓, ROCK↓	Wu et al. (2017a)

Tetramethylpyrazine, extracted from the traditional herbal medicine *Ligusticum*, effectively prevents and reverses PAH in different animal models such as chronic hypoxia-induced PAH, sugen/hypoxia-induced PAH, and MCT-induced PAH in rodents (Chen et al., 2020c). In hypoxia-induced PAH, tetramethylpyrazine improved pulmonary vascular leakage and attenuated the elevation of ROS, HIF1α, and VEGF protein levels (Zhang et al., 2011). Besides, the protective effects on PAH were verified in MCT-induced PAH mainly through inhibiting the proliferation and inflammation of PASMCs, specifically by regulating the activation of the PI3K/Akt signaling pathway (Huang et al., 2021). Furthermore, Cao et al. have previously demonstrated that tetramethylpyrazine could be a worthwhile therapeutic agent in treating PAH dogs induced by acute hypoxia through the induction of plasma ET-1 levels (Cao et al., 1998). Nowadays, the clinical trial of tetramethylpyrazine in PAH treatment is ongoing (registered with www.chictr.org.cn as ChiCTR-IPR-14005379). Therefore, tetramethylpyrazine has the potential to be a novel and inexpensive medication for PAH (Chen et al., 2020c).

Tetrandrine, sourced from *Stephania tetrandra* S. Moore, has various pharmacological effects, including antihypertensive, anticancers, and anti-asthmatic activities (Bhagya and Chandrashekar, 2016; Lin et al., 2019; Luan et al., 2020). A recent study suggested that tetrandrine displayed a preferential vasodilator effect on PAH (Kwan and Wang, 1993; Kim et al., 1997). Tetrandrine inhibited calcium agonist-induced contractile responses with depression of the maximal contraction of PA rings to a varying extent (Wang et al., 2002). In addition, tetrandrine attenuated MCT-induced PAH by regulating NO signaling pathway, antioxidant, and antiproliferation effects (Wang X. et al., 2016).

Berberine, a natural component extracted from the herbal plant *Rizoma Coptidis*, has been applied in various diseases due to its multiple biochemical and pharmacological activities such as antiviral (Warowicka et al., 2020), anti-inflammatory (Ehteshamfar et al., 2020), antitumor (Gu et al., 2020), and cardioprotective effects (Feng X. et al., 2019). Recent studies have suggested that the protective effect of berberine against hypoxia-induced PAH may be achieved mostly through regulating the BMPR2 and TGF-β signaling pathway (Chen M. et al., 2019) and inhibiting the Trx1/β-catenin signaling pathway (Wande et al., 2020). In addition, another study has demonstrated that norepinephrine-induced proliferation and migration of PASMCs can be decreased by berberine mainly through the PP2A signaling pathway, and this therapeutic effect was confirmed in PAH patients and PAH models (Luo et al., 2018). However, berberine has not been clinically studied, and its therapeutic effect and toxicity on PAH need to be further verified.

Betaine, a highly important alkaloid extracted from *Lycium barbarum* THM, possesses numerous pharmacological activities, including anti-inflammatory, antifibrosis, and antioxidation (Zhao et al., 2018). Notably, many studies have found that betaine possesses outstanding anti-inflammatory abilities against a series of inflammatory diseases, such as MCT-induced PAH, and the inflammatory factors, including NF-κB, TNF-α, and IL-1β. The levels of MCP-1 and ET-1 were decreased when treated with betaine (Yang et al., 2018). Since betaine is also used as a dietary supplement, it could be a potentially promising medicine for preventing or alleviating symptoms for PAH patients.

Isorhynchophylline, a tetracyclic oxindole alkaloid isolated from the THM *Uncaria rhynchophylla*, has been used clinically to treat cardiovascular and cerebrovascular diseases (Yang W. et al., 2020; Qin et al., 2021). The effect of isorhynchophylline on PAH was explored due to its antioxidant, anti-inflammatory, anticoagulation, and antiproliferation activities. The results suggested that isorhynchophylline could inhibit PASMCs proliferation *via* suppression of PDGF-Rβ phosphorylation and attenuate pulmonary remodeling after MCT induction (Guo et al., 2014).

Oxymatrine, one of the primary active components of the THM *Sophora flavescens* Ait., exerts positive pharmacological effects such as anti-inflammatory, antioxidant, and antiviral effects, inhibiting the immune reaction, and activity against hepatic fibrosis (Lan et al., 2020). Pharmacological research showed that oxymatrine could prevent PAH through its antiproliferative activity in PASMCs and anti-inflammatory and antioxidant effects in hypoxia- or MCT-induced PAH animal models (Zhang B. et al., 2014).

Aloperine, a quinolizidine alkaloid extracted from THM *Sophora flavescens* Ait., has protective effects on the cardiovascular system. Its positive effect on PAH was demonstrated in MCT-induced PAH rats, which is partially related to RhoA/ROCK pathway (Wu F. et al., 2017). In addition, it negatively regulated NF-κB signaling pathway activity and suppressed HPASMCs proliferation *in vitro* (Chang et al., 2019).

Previous studies have revealed that PAH could be prevented or treated in experimental models by other alkaloids such as α-solanine, colchicine, and capsaicin. Administration of α-solanine decreases distal pulmonary artery remodeling in both MCT-induced and sugen/hypoxia-induced PAH, with possible mechanisms demonstrated in *in vitro* experiments such as AXIN2/β-catenin axis and Akt/GSK-3α pathway (Nie et al., 2017). The inflammatory response was significantly inhibited and the vascular remodeling was alleviated in the MCT-induced PAH animal models when treated with capsaicin or colchicine (Lee et al., 2013; Xu et al., 2017). Capsaicin pretreatment prevented PAH mainly through the p38MAPK pathway (Xu et al., 2017), and colchicine treatment reversed PAH through multiple effects, including inhibiting inflammatory factors, promoting apoptosis, and suppressing the fibrotic biomarkers (Lee et al., 2013).

#### 2.3.3 Traditional Herbal Medicine-Derived Phenolic Acids

Phenolic or phenol carboxylic acids are one of the main classes of plant phenolic compounds, which contain phenolic acids, simple phenols, coumarins, hydrolysable tannins, and stilbenes, and lignins are the most abundant secondary metabolites with one or more hydroxyl groups attached directly to the aromatic ring. They are considered the foremost agents responsible for the biological functions and disease cure (Kumar and Goel, 2019). Notably, phenolic acids are strong natural antioxidants and possess a variety of functions, including anti-inflammatory, antimicrobial, anticancer, antiallergic, antiviral, and antithrombotic effects (Pacheco-Ordaz et al., 2018; Wang J. et al., 2019; Abotaleb et al., 2020; Cardoso et al., 2020). More recently, phenolic acids have attracted interest as a novel therapeutic agent for preventing and treating PAH. The phenol acids from THM with specifically curative effect for the treatment of PAH, such as resveratrol, paclitaxel, salvianolic acid A, magnesium lithospermate B, and osthole, and the identified mechanisms of treating PAH are summarized in Table 5 and their chemical structures in Figure 3C.

**TABLE 5:** Phenolic acids as THM-derived active components for PAH treatment.

Active components	Experimental model	Dose (mg/kg body weight)	Cellular targets	Mechanisms identified	References
Resveratrol	Hypoxia-induced PAH Hypoxic PASMCs	—	PASMCs	• PI3K↑, *p*-Akt/Akt↓	Chen et al. (2014), Xu et al. (2016), Guan et al. (2017), Yu et al. (2017), Li et al. (2020a)
		—	PASMCs	• P21↑, P27↑, MMP9↓, MMP2↓, p-PI3K/PI3K↓, *p*-Akt/Akt↓	
		• 25	—	• SIRT1↑	
		• 40	PASMCs	• p-STAT3↓, *p*-MYPY1↓, RhoA↓, GEF-H1↓	
		• 40	—	• IL-6↓, TNF-α↓, IL-1β↓, VEGF↓, GSH↑, SOD↑, WST-1↓, HIF1α↓, *p*-Akt↓, *p*-ERK↓, Nrf1↑, Nrf2↑, Trx-1↑	
	MCT-induced PAH hypoxic PASMCs	• 30	—	• Cardiomyocyte apoptosis	Csiszar et al. (2009), Yang et al. (2010), Paffett et al. (2012), Wilson et al. (2016), Chen et al. (2020d), Liu et al. (2020d), Vazquez-Garza et al. (2020)
		• 25	PASMCs	• TNFα↓, IL-1β↓, IL-6↓, PDGFα/β↓, eNOS↑, NADPH↓	
		• 3	PASMCs	• atrogin-1↑	
		• 25	PASMCs	• miR-638↑, NR4A3↓, Cyclin D1↓	
		• 25	—	• Inhibits ventricular dysfunction and pathological remodeling changes	
Paclitaxel	MCT-induced PAH	• 2	—	• p27^Kip1^↑, cyclin B1↑	Feng et al. (2019b), Zhao et al. (2019)
		• 5	PASMCs	• p-FoxO1↓, nuclear FoxO1↑, p- FoxO1/t- FoxO1↓, LC3B-II↓, Beclin1↓	
	Schistosoma mansoni-induced PAH	• 25	—	• IL-4↓, IL-13↓, IL-17F↓, TGF-β↓	Kassa et al. (2019)
Salvianolic acid A	MCT-induced PAH	• 0.3, 1, 3	—	• AST↓, ALT↓, LDH↓, NT-proBNP↓, ET-1↓, Bax/Bcl-2↓, BMPR2↑, Smad1/5↑	Chen et al. (2016b), Chen et al. (2017)
		• 0.3, 1, 3	PAECs	• ROS↓, TGF-β1↓, RhoA↓, *p*-Cdc42↓, *p*-Cofilin↓, Nrf2↑, HO-1	
	Hypoxia-induced EndoMT in HPAECs	—	PAECs	• CD31↑, ROS↓, *p*-Smad1/5↑, *p*-Smad2/3↓, *p*-ERK↓, *p*-cofilin↓	Yuan et al. (2017b)
Magnesium lithospermate B	Hypoxia-induced PAH	• 5, 15	PASMCs	• α-SMA↑, SM 22α↑, OPN↓, Cyclin D1↓	(Li et al., 2019a; Li et al., 2019b)
				• NOX2↓, NOX4↓, VPO1↓, HOCl↓, *p*-ERK↓	
Osthole	MCT-induced PAH	• 10, 20	—	• p-NF-κB p65↓, IκBα/β↑, TNF-α↓, COX-2↓, IL-1β↓, IL-6↓	Li et al. (2017), Yao et al. (2018), Yue et al. (2020)
		—	—	• RPL15, Cathepsin S, Histone H3.3, HMGB1	
	Hypoxic PASMCs	—	PASMCs	• TGF-β1↓, *p*-Smad2↓, *p*-Smad3↓, p-P38/P38↓, cyclin D1↓, CDK4↓, cyclin E1↓, CDK2↓, p53↑, p27↑, p21↑)	

Over the past two decades, increasing attention has been given to resveratrol (3,5,4′-trihydroxystilbene), which is a dietary phytoalexin compound with various pharmacological actions such as anti-inflammatory, antioxidant, antiproliferative, and antifibrotic (Chudzińska et al., 2020). Importantly, the multiple actions attributed to resveratrol on the systemic and cardiac vasculature may also target the mediators of PAH (Chicoine et al., 2009). In recent studies, the remarkable efficacy of resveratrol in preventing and treating the PAH has been demonstrated in experimental animal models of PAH induced by hypoxia and MCT (Csiszar et al., 2009; Yang et al., 2010; Paffett et al., 2012; Chen et al., 2014; Wilson et al., 2016; Xu et al., 2016; Guan et al., 2017; Yu et al., 2017; Li C. et al., 2020; Chen et al., 2020d; Liu YY. et al., 2020; Vazquez-Garza et al., 2020). In the hypoxia-induced PAH model, resveratrol has protective effects mainly through preventing PASMC arginase II induction and proliferation that is mediated by the PI3K-Akt signaling pathway (Chen et al., 2014; Guan et al., 2017), enhancing the activation of SIRT1 (Yu et al., 2017), inhibiting the differentiation of Th17 cells (Li C. et al., 2020), suppressing HIF1α expression, and upregulating the Nrf-2/Trx-1 signaling pathway (Xu et al., 2016). In MCT-induced PAH, resveratrol also significantly alleviated the proliferation in PASMCs and vascular remodeling of PAH by improving swollen mitochondrial and cardiomyocyte apoptosis in RV (Yang et al., 2010), inhibiting inflammation and oxidative stress (Csiszar et al., 2009), promoting decreased atrogin-1 levels induced by MCT (Paffett et al., 2012), and targeting NR4A3/cyclin D1 pathway regulated by miR-638 (Liu YY. et al., 2020). In addition, a systematic analysis on the molecular mechanism of resveratrol for treating PAH was recently reported, which showed that the effect might be closely associated with targets, such as AKT1, MAPK3, SIRT1, and SRC, and biological processes, such as cell proliferation, inflammatory response, and redox balance (Chen et al., 2020d). In summary, multiple beneficial effects of resveratrol via multiple targets have been reported in experimental animal models. However, additional preclinical studies followed by clinical trials are essential before resveratrol can be considered a magic bullet for PAH (Chicoine et al., 2009).

Paclitaxel, an antiproliferative drug, has been used to prevent and treat cardiovascular diseases (Baumgartner and Schindewolf, 2020) and has been approved by the U.S. Food and Drug Administration to be used for the prevention of drug-eluting stent-induced restenosis (Leopardi et al., 2014). Given the excessive PASMC proliferation as one of the main characteristics of PAH, researchers have applied paclitaxel to PAH, another vascular disease, and explored its action mechanisms. The results suggested that paclitaxel has positive effects on MCT-induced PAH in rats, which may be associated with the increased expression of p27^Kip1^ and decreased expression of cyclin B1 (Zhao et al., 2019), or suppressed FoxO1-mediated autophagy (Feng et al., 2019b). Moreover, paclitaxel was applied to another PAH mouse model induced by *Schistosoma mansoni* and obtained satisfactory effect by blocking proximate Th2 inflammation rather than suppressing distal TGF-β activation (Kassa et al., 2019).

Salvianolic acid A, a polyphenol extracted from *Salvia miltiorrhiza* Bge., was found to possess pharmacological effects such as cardiovascular protective effects (Wu et al., 2020), nervous system protective effects (Xu et al., 2020), and anti-diabetes effects (Zhou et al., 2020). Recently, the therapeutic effects on PAH by salvianolic acid A were investigated, and it was found that it exhibited a potent inhibitory effect on vascular remodeling through activating the BMPR2-Smad pathway (Chen YC. et al., 2016) and inhibiting apoptosis and endothelial-to-mesenchymal transition (EndMT) (Yuan T. et al., 2017; Chen et al., 2017). Therefore, the treatment of polyphenolic natural antioxidants may be an effective way to attenuate PAH.

Magnesium lithospermate B is an active component of the water-soluble fraction of *Salvia miltiorrhiza*, which possesses pharmacological activities such as anti-inflammation (Gao et al., 2019; Qu et al., 2020), antioxidation (Liu YL. et al., 2019), anti-excitotoxicity (Yang et al., 2015), and anti-apoptosis (Wang M. et al., 2019). Nowadays, researchers have investigated the therapeutic effect on hypoxia-induced PAH and determined that magnesium lithospermate B might be a potent drug for PAH through suppressing NOX/ROS/ERK pathway (Li et al., 2019a) and NOX/VPO1 pathway (Li et al., 2019b). However, its therapeutic effect on PAH needs to be verified in more models to solidify its potential value in the clinic.

Osthole, which belongs to the secondary metabolites of phenolic acids, is coumarin and a principal component isolated from THM *Cnidium monnieri* (L.). Previous studies have revealed that osthole has the pharmacological activities of anti-inflammation, regulating cell cycle, and antiproliferative and vasodilative properties, and its positive effects on PAH have been demonstrated in animal models induced by MCT and in PDGF-BB-induced rat PASMC growth *in vitro* (Li et al., 2017; Yue et al., 2020). Osthole can suppress the progression of vascular remodeling in MCT-induced PAH mediated through modulation of the NF-κB p65 signaling pathway (Li et al., 2017). *In vitro*, the positive effect partly contributed to the downregulation of the TGF-β1/Smad/p38 signaling pathway (Yue et al., 2020). Moreover, a global proteomics study deciphered the novel function of osthole against PAH, which identified the most related target proteins as RPL15, Cathepsin S, Histone H3.3, and HMGB1 (Yao et al., 2018). Therefore, osthole is purposed to be a candidate for new drug development to prevent or treat PAH.

Notably, based on their anti-inflammatory, antioxidant, and anti-proliferative properties, more phenolic acids are explored in preclinical studies for the treatment of PAH. For example, honokiol alleviates MCT-induced PAH by reducing the expression of CyPA and autophagy markers (Wang X. et al., 2019); magnolol ameliorates pneumonectomy and MCT-induced PAH in rats through inhibition of Akt/ERK1/2/GSK3β/β-catenin pathway (Chang et al., 2018); paeonol ameliorated the hypoxia-induced PASMC proliferation in an ERK1/2-dependent manner (Zhang L. et al., 2018); apple polyphenol reversed pulmonary vasoconstriction through enzyme expression and cation channel activities, thus has effects of PASMC relaxation and PAEC protection (Hua et al., 2018); carvacrol attenuated the pulmonary vascular remodeling by partly promoting PASMC the intrinsic apoptotic pathway (Zhang et al., 2016); and schisandrin B mediated vascular relaxation evoked by a direct effect on PASMCs via TGF-β1downstream signal pathways (Wu J. et al., 2017). Moreover, chlorogenic acid, a widely distributed acid, inhibited hypoxia- and MCT-induced PAH via regulating the c-Src-mediated signaling pathway (Li QY. et al., 2015). Ursolic acid exerted beneficial effects on PAH-induced RV dysfunction and remodeling by regulating PPARα-dependent fatty acid metabolism (Gao X. et al., 2020). Ellagic acid ameliorated MCT-induced PAH via exerting the antioxidative property inhibiting NLRP3 inflammasome signal pathway in rats (Tang et al., 2015).

#### 2.3.4 Traditional Herbal Medicine-Derived Glycosides

Glycosides are naturally occurring substances and have strong therapeutic potential and clinical utility (Khan et al., 2019). In treating PAH, numerous glycosides from THM, such as astragaloside IV, salidroside, asiaticoside, ginsenoside Rb1, and notoginsenoside R1, possess outstanding effects, which have one or multi-targets hits during the process of PAH pathogenesis in hypoxia- or MCT-induced experimental models. The identified mechanisms of the frequently studied glycosides are summarized in Table 6, and their chemical structures are depicted in Figure 3D.

**TABLE 6:** Glycosides as THM-derived active components for PAH treatment.

Active components	Experimental model	Dose (mg/kg body weight)	Cellular targets	Mechanisms identified	References
Astragaloside IV	Hypoxia-induced PAH	• 10, 50	PASMCs	• ET-1↓, Ang II↓, TNF-α↓, IL-6↓	Zhang et al. (2018d), Yao et al. (2021)
		• 2	—	• PCNA↓, Jagged-1↓, Notch-3↓, Hes-5↓	
	MCT-induced PAH	• 40, 80	PAECs	• NLRP-3↓, caspase-1↓, ASC↓, IL-18↓, IL-1β↓, calpain-1↓	Sun et al. (2021)
Salidroside	Hypoxia-induced PAH	• 16, 32, 64	PASMCs	• A2aR↑	Huang et al. (2015), Chen et al. (2016a)
		• 2, 8, 32	PASMCs	• PCNA↓, *p*-AMPKα1↑, AMPKα1↑, P53↑, P27↑, P21↑, Bax/Bcl-2↑, Cl-caspase 9↑, Cl-caspase 3↑	
Asiaticoside	Hypoxia-induced PAH	• 50	PASMCs	• TGF-β1↓, *p*-Smad2/3↓	Wang et al. (2015b), Wang et al. (2018b)
		• 50	PAECs	• ET-1↓, NO↑, cGMP↑, *p*-Akt/Akt↑, *p*-eNOS/eNOS↑	
Ginsenoside Rb1	Hypoxic PASMCs	—	PASMCs	• SOCE↓	Jiang et al. (2007), Wang et al. (2015a)
	MCT-induced PAH	• 10, 40	—	• CaN↓, NFAT_3_↓, GATA_4_↓	
Notoginsenoside R1	Hypoxia–hypercapnia-induced pulmonary vasoconstriction	• 8, 40, 100	PASMCs	• p-ERK/t-ERK↓, ERK1↓, ERK2↓	Xu et al. (2014)

Astragaloside IV, a high-purity component extracted from *Astragalus membranaceus*, has been confirmed to possess various pharmacological effects, including antioxidative, anti-inflammatory, cardioprotective, and anticancer (Zhang J. et al., 2020; Wei et al., 2020; Yin et al., 2020). Recent studies have investigated the anti-inflammation effect of astragaloside IV on hypoxia- or MCT-induced PAH in rats and suggested that it decreases the levels of ET-1, Ang II, TNF-α, and IL-6 in hypoxia-induced PAH (Zhang X. et al., 2018) and attenuates the vascular remodeling in experimental PAH through inhibiting the NLRP-3/calpain-1 pathway (Sun et al., 2021). Additionally, astragaloside IV treatment suppressed the expression levels of Jagged-1, Notch-3, and Hes-5 and then inhibited the pulmonary vascular remodeling *in vitro* and *in vivo* mainly through the Notch signaling pathway (Yao et al., 2021). Therefore, astragaloside IV affects multiple targets, including NLRP-3 inflammasome, calpain-1, and Notch-3, to reverse PAH.

As a medicinal plant with broad application prospects, the ability of *Rhodiola* to treat PAH in rodents has been proven (Kosanovic et al., 2013). However, due to the unclear material basis, the mechanical actions of *Rhodiola* in the treatment of PAH are limited. For this reason, in-depth studies were focused on salidroside, the pivotal active ingredient in *Rhodiola*, for its therapeutic effects and pharmacological mechanisms in PAH. Huang et al*.* found that salidroside attenuated hypoxia-induced PAH by elevating PASMCs apoptosis *via* an A2aR-related mitochondria-dependent pathway (Huang et al., 2015). Additionally, salidroside inhibited PASMCs proliferation induced by chronic hypoxia *via* AMPKα1-P53-P27/P21 pathway and reversed apoptosis resistance *via* AMPKα1-P53-Bax/Bcl-2-caspase 9-caspase 3 pathway (Chen M. et al., 2016). Hence, the regulation of apoptosis is believed to be the key mechanism of salidroside in treating chronic hypoxia-induced PAH.

Asiaticoside is a saponin monomer isolated from THM *Centella asiatica*, which has a therapeutic effect on hypoxia-induced PAH in rats. It is mainly through blocking TGF-β1/SMAD family member 2/3 signaling, inducing apoptosis in PASMCs (Wang XB. et al., 2015), promoting NO production mediated by enhancing the phosphorylation of serine/threonine-specific protein kinase/eNOS, and preventing apoptosis in endothelial cells (Wang X. et al., 2018).

Ginsenoside Rb1, the bioactive ingredients of roots from *Panax ginseng*, is one of the most frequently studied natural ingredients with multiple pharmacological effects, including anti-inflammation (Gao H. et al., 2020), neuroprotection (Chen H. et al., 2020), antiviral (Kang et al., 2021), and myocardial protection (Li CY. et al., 2020). Ginsenoside Rb1 is also one of the components in total ginsenosides. Previous research studies confirmed that total ginsenoside is effective in improving the MCT-induced PAH and its secondary RHF, mainly through the antioxidative effect (Qin et al., 2008; Qin et al., 2016). In addition, ginsenoside Rb1 attenuated ET-1-induced contractile response *via* increasing pulmonary vasodilation (Wang RX. et al., 2015). Besides, ginsenoside Rb1 also alleviates cardiac hypertrophy induced by MCT *via* regulating the expressions of cardiac hypertrophic biomarkers such as CaN, NFAT_3_, and GATA_4_ (Jiang et al., 2007). In general, ginsenoside Rb1 improves PAH and its secondary RHF through the joint regulation of the lung and heart, supporting it as a promising new drug worthy of further study.

Notoginsenoside R1, a major bioactive saponin extracted from THM *Panax notoginseng*, exhibits potent anti-inflammatory, neuroprotective, anti-ischemia-reperfusion injury characteristics and anti-apoptosis properties (Guo et al., 2019). Recent research studies hypothesized that notoginsenoside R1 attenuated the activation of the ERK pathway, further inhibiting the hypoxia-hypercapnia-induced vasoconstriction (Xu et al., 2014). Subsequently, Zhao et al. reported the preventive effect of *Panax notoginseng* saponin injection *via* regulating the p38MAPK pathway on a rat model of hypoxia-induced PAH (Zhao et al., 2015).

Recently, the elucidation of the mechanisms of PAH by various components has also become the main direction of THM researchers. For example, glycosides, including polydatin, paeoniflorin, echinacoside, and punicalagin, have prevention and treatment effects on hypoxia-induced PAH in rodents (Miao et al., 2012; Shao et al., 2016; Gai et al., 2020; Yu et al., 2020). Among them, polydatin reverses remodeling through suppressing the expression of PKC, increasing the level of NO, and decreasing the Ang II and ET contents (Miao et al., 2012); paeoniflorin ameliorates BMPR2 downregulation-mediated EndMT and thereafter improves Su5416/hypoxia-induced PAH in rats (Yu et al., 2020); echinacoside has a beneficial effect on hypoxia-induced PAH through regulating PA endothelium and smooth muscle function (Gai et al., 2020). Punicalagin treatment protects against hypoxia-induced endothelial dysfunction and PAH in rats *via* antioxidant actions (Shao et al., 2016). In addition, ruscogenin also exerts dose-dependent effects on MCT-induced PAH through the inhibition of the NF-κB signaling pathway.

At present, numerous studies focused on the PAH treatment by glycosides have shown that they exert therapeutic effects by affecting inflammation, oxidative stress, proliferation, cell apoptosis, cell cycle metastasis, and ion channels. Among them, anti-inflammation and antioxidation are the most prominent, which are mainly achieved by suppressing the NLRP-3 signal and inhibiting the TGF-β1/Smad2/3 signaling and ERK signaling pathways.

#### 2.3.5 Other Active Components Derived From Traditional Herbal Medicine

In addition to the four types of active components derived from THM reviewed above, other types of THM ingredients were also reported on their effects treating PAH. For example, volatile oil, terpenes, and polysaccharides from THM also contain potential active ingredients against the development of PAH.

Basigin (Bsg), a transmembrane glycoprotein, promotes cell proliferation, myofibroblast differentiation, and matrix metalloproteinase activation. Cyclophilin A (CyPA) binds to its receptor Bsg and elevates PASMCs proliferation and inflammatory cell recruitment. The latest study reported that celastrol could be a novel drug for PAH and associated RHF in models induced by MCT, hypoxia, or SU5416/hypoxia that inhibits the expression of Bsg and CyPA (Kurosawa et al., 2021). Triptolide, which has the same source as celastrol, also has a beneficial effect on MCT-induced PAH by promoting regression of PA neointimal formation (Faul et al., 2000). Interestingly, dihydroartemisinin, an artemisinin derivative used to treat malaria, also possesses an excellent effect on treating MCT-induced PAH, mainly through downregulating β-catenin and upregulating the Axin2/GSK-3β, further attenuating the vascular remodeling (Tang et al., 2020). Moreover, it inhibits proliferation, migration, and oxidative stress in hypoxia-induced HPAECs, and autophagy is believed to be the underlying mechanism (Yu et al., 2018). Representative terpene saponins, including glycyrrhizin, 18β-glycyrrhetinic acid, all isolated from the widely used THM *Glycyrrhiza uralensis* Fisch.*,* alleviate the MCT-induced PAH in rats. As the inhibitor of high mobility group box-1, glycyrrhizin attenuates the pulmonary vascular remodeling and PAH (Yang et al., 2014). Similarly, 18β-glycyrrhetinic acid improves the MCT-induced PAH by inhibiting oxidative stress in rats (Zhang M. et al., 2019).

Recently, astragalus polysaccharides have been shown to significantly improve MCT-induced PAH by inhibiting the NF-κB signaling pathway, promoting eNOS synthesis and the secretion of NO, and further improving pulmonary vascular remodeling (Yuan LB. et al., 2017). In short, although the activity of the natural antioxidant polysaccharides against PAH was reported, their mechanism is still undefined.

## 3 Mechanisms of Traditional Herbal Medicine-Derived Active Components for Pulmonary Arterial Hypertension Treatment in Rodents

**FIGURE 4:**
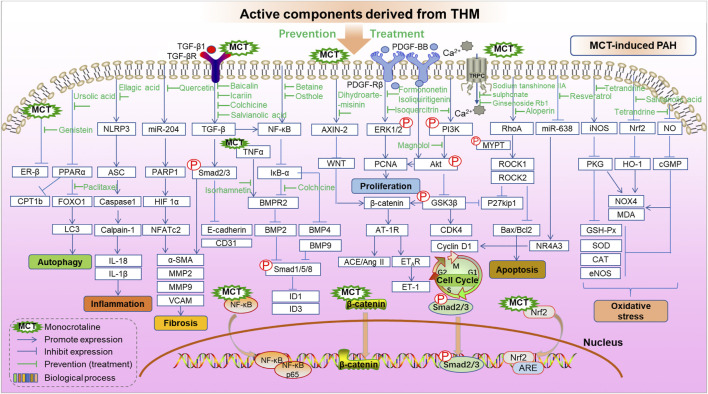
Role and mechanisms of frequently studied active components derived from THM in treating MCT-induced PAH.

**FIGURE 5:**
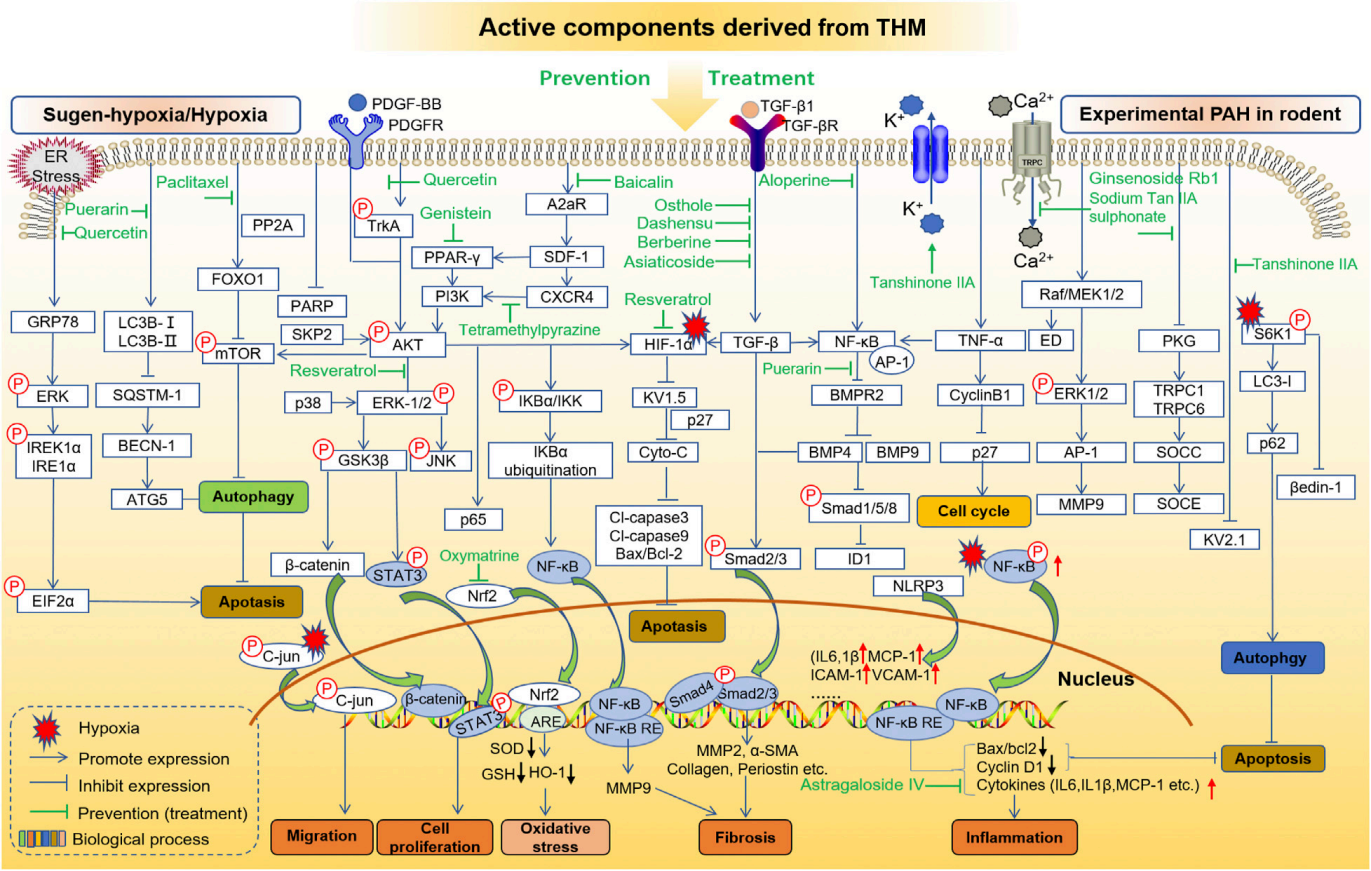
Role and mechanisms of frequently studied active components derived from THM in treating sugen-hypoxia/hypoxia-induced PAH.

MCT, hypoxia, and sugen/hypoxia-induced PAH are the main classic models for preclinical PAH studies. The PAH pathogenesis combined with chemical information of THM yields potential molecular targets of treating PAH, and the *in vivo* and/or *in vitro* effects of these targets can be further investigated. Here, in order to clarify the interaction between the key targets of THM in the treatment of PAH and to provide ideas for the discovery of further combination drugs and new therapeutic targets, the behaviors of active components of THM in the treatment of MCT-, hypoxia/sugen-, and hypoxia-induced PAH are shown, respectively, in Figures 4, 5 **.** As shown in Figure 4, the mechanisms of different THM-derived active compounds in the MCT-induced PAH mouse model are summarized. For example, ursolic acid alleviates PAH by regulating the PPARα-FoxO1-LC3 signaling pathway and inhibiting autophagy. For fibrosis-related pathways, quercetin inhibits the miR-204-PARP1-HIF-α pathway to reduce the expression of α-SMA, MMP2, and other fibrosis-related proteins. Baicalin, icariin, colchicine alkali, and salvianolic acid inhibit the expression of fibrosis-related proteins by inhibiting the TGF-β-smad2/3 pathway. Formononetin and isolicorice inhibit the PDGF-BB-ERK1/2-PCNA signaling pathway to regulate the excessive proliferation of PASMCs. Tetrandrine suppresses the expression of oxidative stress-related factors glutathione peroxidase, superoxide dismutase, catalase, and endothelial nitric oxide synthase to relieve pulmonary hypertension. Mechanisms for hypoxia-induced PHA are summarized in Figure 5, illustrating that THM-related active components inhibit cell autophagy, apoptosis, cycle, and other processes, then affect cell migration, smooth muscle cell proliferation, oxidative stress, fibrosis, and inflammation, and finally inhibit the occurrence and development of pulmonary hypertension. For instance, osthole, dashensu, berberine, and asiaticoside inhibit the phosphorylation of samd2/3 by inhibiting TGF-β, thereby inhibiting pulmonary fibrosis, whereas quercetin inhibits the phosphorylation of ERK by inhibiting GPR78, thereby affecting the cell apoptosis to relieve PAH.

## 4 Lessons Learned From Traditional Chinese Medicine Theory and Future Challenges

Although numerous preclinical and clinical studies of THM have proved their efficacy against PAH, large multi-center clinical trials are still recommended and expected. There are many challenges and difficulties in conducting clinical trials for THM. First, the material basis of THM is less clear, making the settings of exposure-response and toxic-effect studies more complex. Secondly, there are obvious differences between experimental rodent models and patients in PAH disease development. In addition, the patient’s medication compliance should be taken into account in the clinical trials. Moreover, since the oral medication is the main approach of THM, the solubility and bioavailability of THM-derived components should be carefully considered in clinical trials (Gambini et al., 2015; Liu et al., 2016).

The advantage of THM in treating cardiovascular diseases (Yang J. et al., 2020) and chronic diseases such as bronchitis (Yang, 2017), pulmonary fibrosis (Zhang et al., 2021), and immune disorders (Liu W. et al., 2020) is convincing since it is less likely to cause drug resistance and has lesser side effects. Notably, the safety evaluation of THM-derived active ingredients is also essential. However, it should be emphasized that alkaloids have shown specific toxicity (Edgar et al., 2015). MCT, a kind of pyrrolizidine alkaloids found in more than 6000 natural pant species worldwide, can induce hepatic cirrhosis megalocytosis, PAH, and RHF (Ghodsi and Will, 1981; Kay et al., 1982). *Aconiti Lateralis Radix Praeparata* and *Fritillariae Thunbergii Bulbus* show certain aggravated cardiotoxicity in the treatment of experimental PAH, which is attenuated by the co-presence of ginseng (Zhuang et al., 2016; Huang et al., 2018). This strategy also proved a TCM theory that certain herbal combinations may serve to “detoxify” in a prescription.

Interestingly, PAH may alter the intestinal flora and fecal microbiota composition (Callejo et al., 2018; Thenappan et al., 2019). Moreover, the composition and metabolism of gut microbiota can be modulated by THM, and conversely, gut microbiota can also transform THM compounds (Feng et al., 2019a). This is consistent with another classic TCM theory that the lung and the intestine are on the outside/inside of each other. Indeed, the THM-derived active components are the medicinal substances of PAH, and the metabolites or secondary metabolites produced by the active ingredients through a series of processes *in vivo* may also have a therapeutic effect on PAH. Some THM directly exert their beneficial effects on lung infection at least in part through intestinal flora or intestinal lung signals (Lu et al., 2019; Lee et al., 2021). However, whether some of the THM’s beneficial effects on PAH could be through the regulation of intestinal flora remains an open question to be explored further. Similarly, whether the THM metabolites in the body influence PAH has also become a new direction for anti-PAH drug development in the future.

**FIGURE 6:**
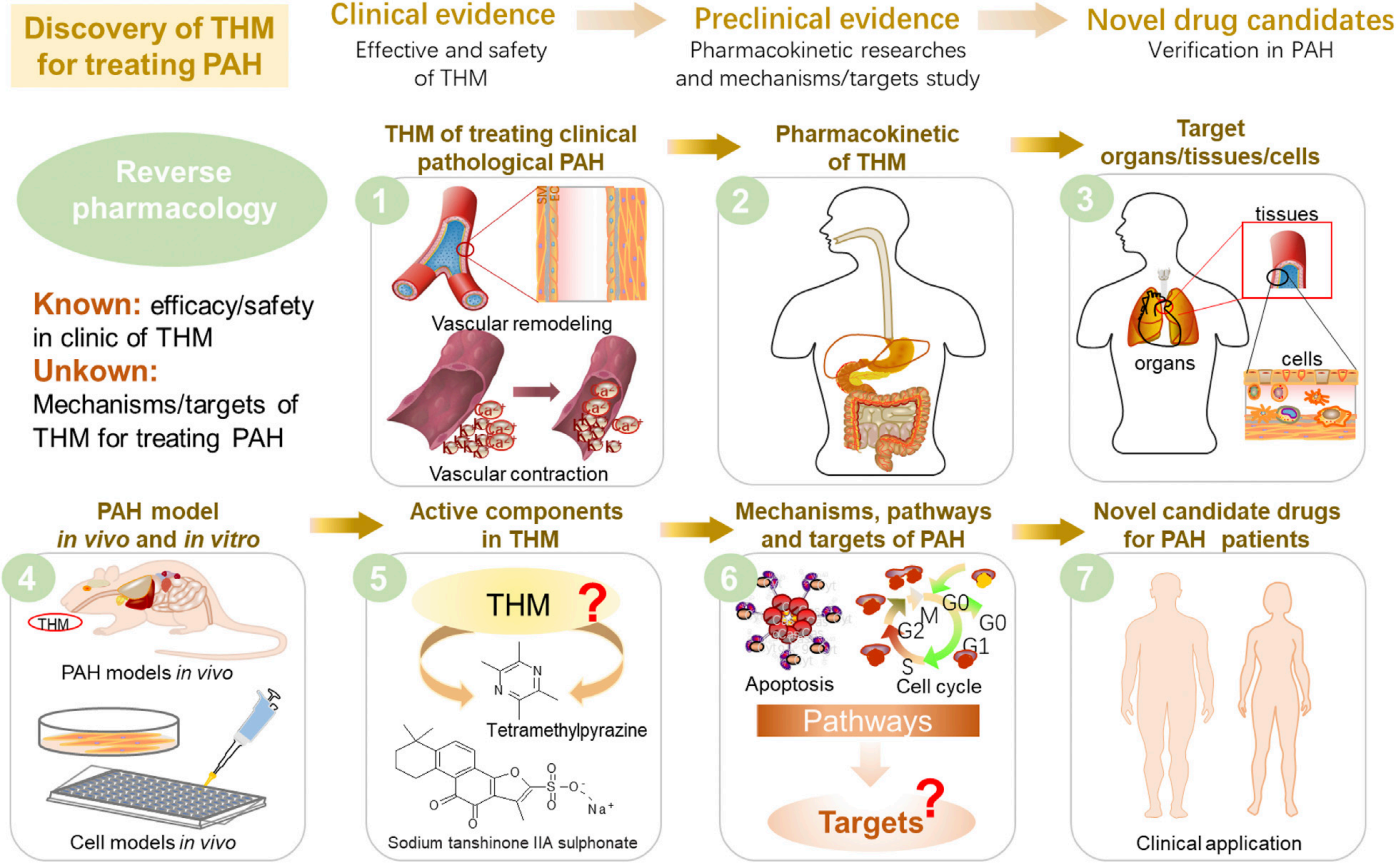
Proposal of reverse pharmacology to discover potential new drug candidates derived from THM that can treat PAH.

It is worth pointing out that reverse pharmacology is a strategy uniquely suited for anti-PAH THM discovery (Figure 6). First of all, the clinical THM collections with high safety and reliable efficacy provided the directions for preclinical research. For instance, the discovery of artemisinin was inspired by the ancient TCM classics books and completed after numerous clinical and experimental tryouts (Tu, 2011). So we can achieve the goal of screening safe and effective drug candidates based on the clinical practices of TCM. Secondly, the identification of THM components and the level of pharmacokinetics in the body are the material basis for post-clinical research. Since there is insufficient evidence to clarify the transformation process and limited understanding of ADME processes of THM, it is difficult to determine which active components play a key role in the pathological process and target changes of the disease (Hao et al., 2014). Therefore, accurate chemical group analysis is currently an important means for THM to find target organs/tissues/cells in the body. At present, the global detection and identification of target or non-target components can be found through a single mass spectrometry analysis. At the same time, the realization of the structural analysis of the components is a quick way to find compounds with similar components. For example, based on the characteristics of PAH ion channel disorder and accumulation of inflammation in lung tissue, the cardiovascular drug tanshinone IIA sodium sulfonate, which is commonly used in clinics to regulate ion channels and inhibit inflammation, has been found to have a better improvement effect on experimental PAH (Huang et al., 2009; Jiang et al., 2016; Bao et al., 2020). Furthermore, the preclinical PAH model was established *in vivo* and *in vitro* to verify the efficacy of the THM. Moreover, the potential targets and pathways can be confirmed through preclinical experiments and molecular biotechnology. Finally, as a potential drug candidate for the treatment of PAH, it must undergo further clinical and large-scale verification.

To verify the efficacy of THM in different PAH models *in vivo*, combined multi-omics technologies, including transcriptomics, proteomics, metabolomics, single-cell sequencing, and 16S sequencing have become emerging tools for tapping the interactive pathways and multiple targets during the THM treatment (He et al., 2017). *In vitro*, the use of omics to perform high-throughput screening of the primary ingredients and tissue-specific metabolites obtained in the early stage has become an important means to consolidate true active ingredients that are most relevant to PAH. Based on the currently identified PAH pathogenic targets or pathways, high-throughput screening of other components with the common nucleus structure can be applied to discover the new drugs. Finally, the established targets of THM components should be explored and verified in the PAH setting to “repurpose” the old drug. At present, clinical trials for sodium tanshinone IIA and sulfonate tetramethylpyrazine have been launched, and successful outcomes will offer novel and natural drugs for treating PAH. The pathogenic mechanism/target and component combinative analysis of PAH will provide further opportunities for THM. The potential targets obtained could be verified through preclinical studies such as animal models and cell models to further guide the development of new drugs.

## 5 Conclusion and Future Perspectives

THM are valuable natural sources for discovering and developing novel drugs for preventing and treating PAH with unique properties. Over half of the current drugs and formulations are derivatives of active components from THM. Currently, most anti-PAH THMs are in the early stage of preclinical research. Compared to the clinical application of the 12 FDA-approved drugs for the treatment of PAH (tadalafil, sildenafil, Riociguat, bosentan, ambrisentan, macitentan, epoprostenol, room-temperature stable epoprostenol, treprostinil, inhaled treprostinil, inhaled iloprost, and oral treprostinil), there is only limited use of THM or a combination of Chinese and Western medicine to improve the complications of PAH patients by TCM physicians, due in part to the lack of large-scale and multi-center RCT evidence for the efficacy and safety.

Although the diagnosis and treatment approaches of Chinese medicine are fundamentally different from modern medicine, evidence-based Chinese medicine and the integration of Chinese medicine principles and medication with modern science and medicine is an area of tremendous ongoing interest and effort (Wang J. et al., 2018). Generally, the role of THM in PAH treatment is weaker than that of chemical drugs. THM is characterized by the individual adjustment of multiple components and multiple targets, enabling the body to transform from an abnormal to normal state, while chemical drugs are primarily for a single target (Xiang et al., 2018). For example, sildenafil and vardenafil, which are approved to treat PAH by FDA, can inhibit phosphodiesterase-5 (PDE-5) and prevent the lungs from breaking down the natural substances that cause blood vessel relaxation. Riociguat can cause pulmonary artery cells to produce more cyclic GMP, which is a substance that relaxes the pulmonary artery. Proved by numerous studies, the efficacy of resveratrol for PAH was attributed, in part, to reduced PASMC proliferation, increased eNOS and NO bioavailability, reduced oxidative stress, decreased inflammatory cytokine levels, and decreased lung leukocyte infiltrate and iNOS levels (Chicoine et al., 2009).”

In this review, we summarize the updated clinical investigation and trials, preclinical findings on the chemical basis, pharmacological efficacies, action targets, and identified mechanisms of THM and their active components for the PAH therapeutics. Moreover, inspired by traditional Chinese medicine theory, a reverse pharmacology strategy is proposed here to guide the bedside-bench-bedside drug discovery and transformation of THM in the PAH setting.

## Glossary

ADAMTS-1: A disintegrin-like and metalloproteinase with thrombospondin type 1vmotif
AhR: Aryl hydrocarbon receptor
Ang II: Angiotensin II
Bax: Bcl2-associated X
Bcl2: B-cell lymphoma-2
BMP: Bone morphogenetic protein
BMPR2: Bone morphogenetic protein receptor 2
CaMKII: Calmodulin-dependent kinase II
CAT: Catalase
CDK2: Cyclin-dependent kinase 2
CDK4: Cyclin-dependent kinase 4
CFDA: China Food and Drug Administration
COX-2: Cyclooxygenase-2
CXCR4: C-X-C motif chemokine receptor 4
CyPA: Cyclophilin A
CytoC: Cytochrome c oxidase
eNOS: Endothelial nitric oxide synthase
EPO: Erythropoietin
EPOR: Erythropoietin receptor
ERK1: Extracellular regulated protein kinase 1
ERK2: Extracellular regulated protein kinase 2
ERS: Endoplasmic reticulum stress
ET-1: Endothelin-1
FDA: U.S. Food and Drug Administration
FoXO1: Forkhead box proteins 1
GATA4: GATA-binding protein 4
GSH: Glutathione synthetase
GSK3β: Glycogen synthase kinase-3β
HIF1α: Hypoxia-inducible factor 1-alpha
HMGB1: High mobility group protein B1
HO-1: Heme oxygenase-1
ICAM-1: Intercellular adhesion molecule-1
IL-10: Interleukin-10
IL-1β: Interleukin-1β
IL-6: Interleukin-6
iNOS: Inducible nitric oxide synthase
vWF: von Willebrand factor
ATG5: Autophagy protein 5
IRE1α: Inositol-requiring enzyme 1α
MAPK: Mitogen-activated protein kinase
MCP-1: Monocyte chemotactic protein-1
MCT: Monocrotaline
MDA: Malondialdehyde
MMP2: Matrix metalloproteinase-2
MMP7: Matrix metalloproteinase-7
MMP-9: Matrix metalloproteinase-9
mTOR: Mammalian target of rapamycin
NADPH: Nicotinamide adenine dinucleotide phosphate
NFAT3: Nuclear factor of activated T cells 3
NFATc2: Nuclear factor of activated T cells cytoplasmic 2
NF-κB: Nuclear factor kappa-B
NLRP-3: Nod-like receptor pyrin containing-3
NO: Nitric Oxide
NOX1: NADPH oxidase 1
NOX4: NADPH oxidase 4
Nrf2: Nuclear factor erythroid 2-related factor 2
p38MAPK: Mitogen-activated protein kinase
PA: Pulmonary artery
PASMCs: Pulmonary artery smooth cells
NOS: Nitric oxide synthase
PAH: Pulmonary arterial hypertension
PARP1: Poly ADP-ribose polymerase-1
PCNA: Proliferating cell nuclear antigen
PDE4D: cAMP-specific 3',5'-cyclic phosphodiesterase 4D
PDGF-Rβ: Platelet-derived growth factor β receptor
PDK1: 3-Phosphoinositide dependent kinase-1
PED5: Phosphodiesterase type-5
PI3K: Phosphoinositide 3-kinase
PKA: Protein kinase A
PKG: Protein kinase G
PP2A: Protein phosphatase 2A
PPAR-γ: Peroxisome proliferator-activated receptor-γ

PASMCs: Pulmonary artery smooth muscle cells
RCT: Randomized controlled trials
RHF: Right heart failure
RhoA: ras homolog family member A
ROCK: Rho-associated coiled-coil containing protein kinase
ROS: Reactive oxygen species
RPL15: 60S ribosomal protein L15
sGC: Soluble guanylyl cyclase
SIRT1: Silent information regulation 2 homolog1
Skp2: S-phase kinase-associated protein 2
Smad3: Smad family member 3
SOCE: Store-operated Ca2+ entry
SOD: Superoxide dismutase
SOD1: Superoxide dismutase 1
SQSTM1: Sequestosome-1
STAT3: Signal transducers and activators of transcription 3
TCM: Traditional Chinese medicines
TGF-β1: Transforming growth factor-β1
THM: Traditional herbal medicine
TNFα: Tumor necrosis factor-alpha
TrkA: Tyrosine receptor kinase A
TRPC1: Short transient receptor potential channel 1
TRPC6: Short transient receptor potential channel 6
VCAM-1: Vascular cell adhesion molecule-1
VEGF: Vascular endothelial growth factor.

## References

[B1] Abotaleb M. Liskova A. Kubatka P. Büsselberg D. (2020). Therapeutic Potential of Plant Phenolic Acids in the Treatment of Cancer. Biomolecules 10 (2), 221. 10.3390/biom10020221 PMC7072661 32028623

[B2] Ahmadipour B. Kalantar M. Hosseini S. Yang L. Kalantar M. Raza S. (2017). Hawthorn (Crataegus Oxyacantha) Extract in the Drinking Water of Broilers on Growth and Incidence of Pulmonary Hypertension Syndrome (Phs). Rev. Bras. Cienc. Avic. 19 (4), 639–644. 10.1590/1806-9061-2017-0558

[B3] Ahmed L. A. Obaid A. A. Zaki H. F. Agha A. M. (2014). Naringenin Adds to the Protective Effect of L-Arginine in Monocrotaline-Induced Pulmonary Hypertension in Rats: Favorable Modulation of Oxidative Stress, Inflammation and Nitric Oxide. Eur. J. Pharm. Sci. 62, 161–170. 10.1016/j.ejps.2014.05.011 24878387

[B4] Alasvand M. Assadollahi V. Ambra R. Hedayati E. Kooti W. Peluso I. (2019). Antiangiogenic Effect of Alkaloids. Oxid Med. Cel Longev 2019, 9475908. 10.1155/2019/9475908 PMC6501137 31178979

[B5] Archer S. L. Weir E. K. Wilkins M. R. (2010). Basic Science of Pulmonary Arterial Hypertension for Clinicians: New Concepts and Experimental Therapies. Circulation 121 (18), 2045–2066. 10.1161/CIRCULATIONAHA.108.847707 20458021 PMC2869481

[B6] Bae H. J. Sowndhararajan K. Park H. B. Kim S. Y. Kim S. Kim D. H. (2019). Danshensu Attenuates Scopolamine and Amyloid-β-Induced Cognitive Impairments through the Activation of PKA-CREB Signaling in Mice. Neurochem. Int. 131, 104537. 10.1016/j.neuint.2019.104537 31425745

[B7] Bao Y. R. Chen J. W. Jiang Y. Wang L. H. Xue R. Qian J. X. (2020). Sodium Tanshinone Ii Sulfonate a Ameliorates Hypoxia-Induced Pulmonary Hypertension. Front. Pharmacol. 11, 687. 10.3389/fphar.2020.00687 32508639 PMC7253651

[B8] Baumgartner I. Schindewolf M. (2020). The Paclitaxel story in Cardiovascular Medicine. Eur. Heart J. 41 (38), 3740–3742. 10.1093/eurheartj/ehz881 31951252

[B9] Bhagya N. Chandrashekar K. R. (2016). Tetrandrine--a Molecule of Wide Bioactivity. Phytochemistry 125, 5–13. 10.1016/j.phytochem.2016.02.005 26899361

[B10] Bombicz M. Priksz D. Varga B. Kurucz A. Kertész A. Takacs A. (2017). A Novel Therapeutic Approach in the Treatment of Pulmonary Arterial Hypertension: Allium Ursinum Liophylisate Alleviates Symptoms Comparably to Sildenafil. Int. J. Mol. Sci. 18 (7), 1436. 10.3390/ijms18071436 PMC5535927 28677661

[B11] Cai C. Xiang Y. Wu Y. Zhu N. Zhao H. Xu J. (2019). Formononetin Attenuates Monocrotaline-induced P-ulmonary A-rterial H-ypertension via I-nhibiting P-ulmonary V-ascular R-emodeling in R-ats. Mol. Med. Rep. 20 (6), 4984–4992. 10.3892/mmr.2019.10781 31702810 PMC6854580

[B12] Callejo M. Mondejar-Parreño G. Barreira B. Izquierdo-Garcia J. L. Morales-Cano D. Esquivel-Ruiz S. (2018). Pulmonary Arterial Hypertension Affects the Rat Gut Microbiome. Sci. Rep. 8 (1), 9681. 10.1038/s41598-018-27682-w 29946072 PMC6018770

[B13] Cao G. Zhu R. Jiang T. Tang D. Kwan H. Y. Su T. (2019a). Danshensu, a Novel Indoleamine 2,3-dioxygenase1 Inhibitor, Exerts Anti-hepatic Fibrosis Effects via Inhibition of Jak2-Stat3 Signaling. Phytomedicine 63, 153055. 10.1016/j.phymed.2019.153055 31377585

[B14] Cao W. Zeng Z. Zhu Y. J. Luo W. Demura H. Naruse M. (1998). Effects of Tetramethylpyrazine, a Chinese Medicine, on Plasma Endothelin-1 Levels during Acute Pulmonary Hypoxia in Anesthetized Dogs. J. Cardiovasc. Pharmacol. 31 Suppl 1 (Suppl. 1), S456–S459. 10.1097/00005344-199800001-00130 9595511

[B15] Cao X. He Y. Li X. Xu Y. Liu X. (2019b). The IRE1α-XBP1 Pathway Function in Hypoxia-Induced Pulmonary Vascular Remodeling, Is Upregulated by Quercetin, Inhibits Apoptosis and Partially Reverses the Effect of Quercetin in PASMCs. Am. J. Transl Res. 11 (2), 641–654. 30899368 PMC6413268

[B16] Cardoso R. R. Neto R. O. Dos Santos D'Almeida C. T. do Nascimento T. P. Pressete C. G. Azevedo L. (2020). Kombuchas from green and Black Teas Have Different Phenolic Profile, Which Impacts Their Antioxidant Capacities, Antibacterial and Antiproliferative Activities. Food Res. Int. 128, 108782. 10.1016/j.foodres.2019.108782 31955755

[B17] Chan E. Tan M. Xin J. Sudarsanam S. Johnson D. E. (2010). Interactions between Traditional Chinese Medicines and Western Therapeutics. Curr. Opin. Drug Discov. Devel 13 (1), 50–65. 20047146

[B18] Chang H. Chang C. Y. Lee H. J. Chou C. Y. Chou T. C. (2018). Magnolol Ameliorates Pneumonectomy and Monocrotaline-Induced Pulmonary Arterial Hypertension in Rats through Inhibition of Angiotensin Ii and Endothelin-1 Expression. Phytomedicine 51, 205–213. 10.1016/j.phymed.2018.10.001 30466619

[B19] Chang Z. Wang J. L. Jing Z. C. Ma P. Xu Q. B. Na J. R. (2020). Protective Effects of Isorhamnetin on Pulmonary Arterial Hypertension: *In Vivo* and *In Vitro* Studies. Phytother Res. 34 (10), 2730–2744. 10.1002/ptr.6714 32452118

[B20] Chang Z. Zhang P. Zhang M. Jun F. Hu Z. Yang J. (2019). Aloperine Suppresses Human Pulmonary Vascular Smooth Muscle Cell Proliferation via Inhibiting Inflammatory Response. Chin. J. Physiol. 62 (4), 157–165. 10.4103/CJP.CJP_27_19 31535631

[B21] Chen B. Xue J. Meng X. Slutzky J. L. Calvert A. E. Chicoine L. G. (2014). Resveratrol Prevents Hypoxia-Induced Arginase Ii Expression and Proliferation of Human Pulmonary Artery Smooth Muscle Cells via Akt-dependent Signaling. Am. J. Physiol. Lung Cel Mol Physiol 307 (4), L317–L325. 10.1152/ajplung.00285.2013 PMC4137162 24951775

[B22] Chen C. Chen C. Wang Z. Wang L. Yang L. Ding M. (2012). Puerarin Induces Mitochondria-dependent Apoptosis in Hypoxic Human Pulmonary Arterial Smooth Muscle Cells. PLoS One 7 (3), e34181. 10.1371/journal.pone.0034181 22457823 PMC3311615

[B23] Chen H. Shen J. Li H. Zheng X. Kang D. Xu Y. (2020a). Ginsenoside Rb1 Exerts Neuroprotective Effects through Regulation of Lactobacillus Helveticus Abundance and GABAA Receptor Expression. J. Ginseng Res. 44 (1), 86–95. 10.1016/j.jgr.2018.09.002 32095096 PMC7033341

[B24] Chen M. Cai H. Yu C. Wu P. Fu Y. Xu X. (2016a). Salidroside Exerts Protective Effects against Chronic Hypoxia-Induced Pulmonary Arterial Hypertension via AMPKα1-dependent Pathways. Am. J. Transl Res. 8 (1), 12–27. 27069536 PMC4759412

[B25] Chen M. Shen H. Zhu L. Yang H. Ye P. Liu P. (2019a). Berberine Attenuates Hypoxia-Induced Pulmonary Arterial Hypertension via Bone Morphogenetic Protein and Transforming Growth Factor-β Signaling. J. Cel Physiol 234 (10), 17482–17493. 10.1002/jcp.28370 30786011

[B26] Chen W. Wang S. Wu Y. Shen X. Xu S. Guo Z. (2020b). The Physiologic Activity and Mechanism of Quercetin-like Natural Plant Flavonoids. Curr. Pharm. Biotechnol. 21 (8), 654–658. 10.2174/1389201021666200212093130 32048963

[B27] Chen Y. Lu W. Yang K. Duan X. Li M. Chen X. (2020c). Tetramethylpyrazine: A Promising Drug for the Treatment of Pulmonary Hypertension. Br. J. Pharmacol. 177 (12), 2743–2764. 10.1111/bph.15000 31976548 PMC7236078

[B28] Chen Y. Yuan T. Chen D. Liu S. Guo J. Fang L. (2020d). Systematic Analysis of Molecular Mechanism of Resveratrol for Treating Pulmonary Hypertension Based on Network Pharmacology Technology. Eur. J. Pharmacol. 888, 173466. 10.1016/j.ejphar.2020.173466 32798507

[B29] Chen Y. Yuan T. Zhang H. Yan Y. Wang D. Fang L. (2017). Activation of Nrf2 Attenuates Pulmonary Vascular Remodeling via Inhibiting Endothelial-To-Mesenchymal Transition: An Insight from a Plant Polyphenol. Int. J. Biol. Sci. 13 (8), 1067–1081. 10.7150/ijbs.20316 28924387 PMC5599911

[B30] Chen Y. C. Yuan T. Y. Zhang H. F. Wang D. S. Yan Y. Niu Z. R. (2016b). Salvianolic Acid a Attenuates Vascular Remodeling in a Pulmonary Arterial Hypertension Rat Model. Acta Pharmacol. Sin 37 (6), 772–782. 10.1038/aps.2016.22 27180980 PMC4954764

[B31] Chen Y. Chen D. Liu S. Yuan T. Guo J. Fang L. (2019b). Systematic Elucidation of the Mechanism of Genistein against Pulmonary Hypertension via Network Pharmacology Approach. Ijms 20 (22), 5569. 10.3390/ijms20225569 PMC6888439 31703458

[B32] Chicoine L. G. Stewart J. A. Jr. Lucchesi P. A. (2009). Is Resveratrol the Magic Bullet for Pulmonary Hypertension? Hypertension 54 (3), 473–474. 10.1161/HYPERTENSIONAHA.109.135251 19597034 PMC2730375

[B33] Chudzińska M. Rogowicz D. Wołowiec Ł. Banach J. Sielski S. Bujak R. (2020). Resveratrol and Cardiovascular System-The Unfulfilled Hopes. Ir J. Med. Sci. 190, 981–986. 10.1007/s11845-020-02441-x 33219913

[B34] Csiszar A. Labinskyy N. Olson S. Pinto J. T. Gupte S. Wu J. M. (2009). Resveratrol Prevents Monocrotaline-Induced Pulmonary Hypertension in Rats. Hypertension 54 (3), 668–675. 10.1161/HYPERTENSIONAHA.109.133397 19597040 PMC2745434

[B35] Edgar J. A. Molyneux R. J. Colegate S. M. (2015). Pyrrolizidine Alkaloids: Potential Role in the Etiology of Cancers, Pulmonary Hypertension, Congenital Anomalies, and Liver Disease. Chem. Res. Toxicol. 28 (1), 4–20. 10.1021/tx500403t 25483859

[B36] Ehteshamfar S. M. Akhbari M. Afshari J. T. Seyedi M. Nikfar B. Shapouri-Moghaddam A. (2020). Anti-inflammatory and Immune-Modulatory Impacts of Berberine on Activation of Autoreactive T Cells in Autoimmune Inflammation. J. Cel Mol Med 24 (23), 13573–13588. 10.1111/jcmm.16049 PMC7754052 33135395

[B37] Ezzati M. Yousefi B. Velaei K. Safa A. (2020). A Review on Anti-cancer Properties of Quercetin in Breast Cancer. Life Sci. 248, 117463. 10.1016/j.lfs.2020.117463 32097663

[B38] Fallon M. B. Abrams G. A. Abdel-Razek T. T. Dai J. Chen S. J. Chen Y. F. (1998). Garlic Prevents Hypoxic Pulmonary Hypertension in Rats. Am. J. Physiol. 275 (2), L283–L287. 10.1152/ajplung.1998.275.2.L283 9700088

[B39] Faul J. L. Nishimura T. Berry G. J. Benson G. V. Pearl R. G. Kao P. N. (2000). Triptolide Attenuates Pulmonary Arterial Hypertension and Neointimal Formation in Rats. Am. J. Respir. Crit. Care Med. 162 (6), 2252–2258. 10.1164/ajrccm.162.6.2002018 11112148

[B40] Feng W. Ao H. Peng C. Yan D. (2019a). Gut Microbiota, a New Frontier to Understand Traditional Chinese Medicines. Pharmacol. Res. 142, 176–191. 10.1016/j.phrs.2019.02.024 30818043

[B41] Feng W. Wang J. Yan X. Zhai C. Shi W. Wang Q. (2019b). Paclitaxel Alleviates Monocrotaline-Induced Pulmonary Arterial Hypertension via Inhibition of Foxo1-Mediated Autophagy. Naunyn Schmiedebergs Arch. Pharmacol. 392 (5), 605–613. 10.1007/s00210-019-01615-4 30683943

[B42] Feng X. Sureda A. Jafari S. Memariani Z. Tewari D. Annunziata G. (2019c). Berberine in Cardiovascular and Metabolic Diseases: From Mechanisms to Therapeutics. Theranostics 9 (7), 1923–1951. 10.7150/thno.30787 31037148 PMC6485276

[B43] Ferenczyova K. Kalocayova B. Bartekova M. (2020). Potential Implications of Quercetin and its Derivatives in Cardioprotection. Int. J. Mol. Sci. 21 (5). 10.3390/ijms21051585 PMC7084176 32111033

[B44] Gai X. Lin P. He Y. Lu D. Li Z. Liang Y. (2020). Echinacoside Prevents Hypoxic Pulmonary Hypertension by Regulating the Pulmonary Artery Function. J. Pharmacol. Sci. 144 (4), 237–244. 10.1016/j.jphs.2020.09.002 33070843

[B45] Gambini J. Inglés M. Olaso G. Lopez-Grueso R. Bonet-Costa V. Gimeno-Mallench L. (2015). Properties of Resveratrol: *In Vitro* and *In Vivo* Studies about Metabolism, Bioavailability, and Biological Effects in Animal Models and Humans. Oxid Med. Cel Longev 2015, 837042. 10.1155/2015/837042 PMC4499410 26221416

[B46] Gao F. Li J. M. Xi C. Li H. H. Liu Y. L. Wang Y. P. (2019). Magnesium Lithospermate B Protects the Endothelium from Inflammation-Induced Dysfunction through Activation of Nrf2 Pathway. Acta Pharmacol. Sin 40 (7), 867–878. 10.1038/s41401-018-0189-1 30617294 PMC6786373

[B47] Gao H. Chen C. Huang S. Li B. (2012). Quercetin Attenuates the Progression of Monocrotaline-Induced Pulmonary Hypertension in Rats. J. Biomed. Res. 26 (2), 98–102. 10.1016/s1674-8301(12)60018-9 23554737 PMC3597325

[B48] Gao H. Kang N. Hu C. Zhang Z. Xu Q. Liu Y. (2020a). Ginsenoside Rb1 Exerts Anti-inflammatory Effects *In Vitro* and *In Vivo* by Modulating Toll-like Receptor 4 Dimerization and Nf-Kb/mapks Signaling Pathways. Phytomedicine 69, 153197. 10.1016/j.phymed.2020.153197 32146298

[B49] Gao X. Zhang Z. Li X. Wei Q. Li H. Li C. (2020b). Ursolic Acid Improves Monocrotaline-Induced Right Ventricular Remodeling by Regulating Metabolism. J. Cardiovasc. Pharmacol. 75 (6), 545–555. 10.1097/fjc.0000000000000815 32141989

[B50] Ghodsi F. Will J. A. (1981). Changes in Pulmonary Structure and Function Induced by Monocrotaline Intoxication. Am. J. Physiol. 240 (2), H149–H155. 10.1152/ajpheart.1981.240.2.H149 6451183

[B51] Gu S. Song X. Xie R. Ouyang C. Xie L. Li Q. (2020). Berberine Inhibits Cancer Cells Growth by Suppressing Fatty Acid Synthesis and Biogenesis of Extracellular Vesicles. Life Sci. 257, 118122. 10.1016/j.lfs.2020.118122 32702446

[B52] Guan Z. Shen L. Liang H. Yu H. Hei B. Meng X. (2017). Resveratrol Inhibits Hypoxia-Induced Proliferation and Migration of Pulmonary Artery Vascular Smooth Muscle Cells by Inhibiting the Phosphoinositide 3-kinase/protein Kinase B Signaling Pathway. Mol. Med. Rep. 16 (2), 1653–1660. 10.3892/mmr.2017.6814 28656233 PMC5562090

[B53] Guo H. Zhang X. Cui Y. Deng W. Xu D. Han H. (2014). Isorhynchophylline Protects against Pulmonary Arterial Hypertension and Suppresses Pasmcs Proliferation. Biochem. Biophys. Res. Commun. 450 (1), 729–734. 10.1016/j.bbrc.2014.06.044 24950404

[B54] Guo S. Xi X. Li J. (2019). Notoginsenoside R1: A Systematic Review of its Pharmacological Properties. Pharmazie 74 (11), 641–647. 10.1691/ph.2019.9534 31739829

[B55] Han B. Che X. Zhao Y. Li C. He J. Lu Y. (2019). Neuroprotective Effects of Danshensu in Parkinson's Disease Mouse Model Induced by 1-Methyl-4-Phenyl-1,2,3,6-Tetrahydropyridine. Behav. Pharmacol. 30 (1), 36–44. 10.1097/fbp.0000000000000412 29847337

[B56] Han C. Qi J. Gao S. Li C. Ma Y. Wang J. (2017). Vasodilation Effect of Volatile Oil from Allium Macrostemon Bunge Are Mediated by Pka/no Pathway and its Constituent Dimethyl Disulfide in Isolated Rat Pulmonary Arterials. Fitoterapia 120, 52–57. 10.1016/j.fitote.2017.05.007 28552597

[B57] Han X. Zhang Y. Zhou Z. Zhang X. Long Y. (2016). Hydroxysafflor Yellow a Improves Established Monocrotaline-Induced Pulmonary Arterial Hypertension in Rats. J. Int. Med. Res. 44 (3), 569–584. 10.1177/0300060515597931 27059291 PMC5536702

[B58] Hao H. Zheng X. Wang G. (2014). Insights into Drug Discovery from Natural Medicines Using Reverse Pharmacokinetics. Trends Pharmacol. Sci. 35 (4), 168–177. 10.1016/j.tips.2014.02.001 24582872

[B59] He Q. Nan X. Li S. Su S. Ma K. Li Z. (2018). Tsantan Sumtang Alleviates Chronic Hypoxia-Induced Pulmonary Hypertension by Inhibiting Proliferation of Pulmonary Vascular Cells. Biomed. Res. Int. 2018, 9504158. 10.1155/2018/9504158 30622966 PMC6304203

[B60] He Y. Cao X. Guo P. Li X. Shang H. Liu J. (2017). Quercetin Induces Autophagy via Foxo1-dependent Pathways and Autophagy Suppression Enhances Quercetin-Induced Apoptosis in Pasmcs in Hypoxia. Free Radic. Biol. Med. 103, 165–176. 10.1016/j.freeradbiomed.2016.12.016 27979659

[B61] He Y. Cao X. Liu X. Li X. Xu Y. Liu J. (2015). Quercetin Reverses Experimental Pulmonary Arterial Hypertension by Modulating the Trka Pathway. Exp. Cel Res 339 (1), 122–134. 10.1016/j.yexcr.2015.10.013 26476374

[B62] He Y. Fang X. Shi J. Li X. Xie M. Liu X. (2020). Apigenin Attenuates Pulmonary Hypertension by Inducing Mitochondria-dependent Apoptosis of PASMCs via Inhibiting the Hypoxia Inducible Factor 1α-KV1.5 Channel Pathway. Chem. Biol. Interact 317, 108942. 10.1016/j.cbi.2020.108942 31930969

[B63] Hoeper M. M. Humbert M. Souza R. Idrees M. Kawut S. M. Sliwa-Hahnle K. (2016). A Global View of Pulmonary Hypertension. Lancet Respir. Med. 4 (4), 306–322. 10.1016/s2213-2600(15)00543-3 26975810

[B64] Hsu W. L. Lin Y. C. Jeng J. R. Chang H. Y. Chou T. C. (2018). Baicalein Ameliorates Pulmonary Arterial Hypertension Caused by Monocrotaline through Downregulation of Et-1 and Etar in Pneumonectomized Rats. Am. J. Chin. Med. 46 (4), 769–783. 10.1142/S0192415X18500404 29737212

[B65] Hua C. Zhao J. Wang H. Chen F. Meng H. Chen L. (2018). Apple Polyphenol Relieves Hypoxia-Induced Pulmonary Arterial Hypertension via Pulmonary Endothelium protection and Smooth Muscle Relaxation: *In Vivo* and *In Vitro* Studies. Biomed. Pharmacother. 107, 937–944. 10.1016/j.biopha.2018.08.080 30257406

[B66] Huang H. Kong L. Luan S. Qi C. Wu F. (2021). Ligustrazine Suppresses Platelet-Derived Growth Factor-Bb-Induced Pulmonary Artery Smooth Muscle Cell Proliferation and Inflammation by Regulating the Pi3k/akt Signaling Pathway. Am. J. Chin. Med. 49 (2), 437–459. 10.1142/s0192415x21500208 33622214

[B67] Huang S. Chen P. Shui X. He Y. Wang H. Zheng J. (2014). Baicalin Attenuates Transforming Growth Factor-Β1-Induced Human Pulmonary Artery Smooth Muscle Cell Proliferation and Phenotypic Switch by Inhibiting Hypoxia Inducible Factor-1α and Aryl Hydrocarbon Receptor Expression. J. Pharm. Pharmacol. 66 (10), 1469–1477. 10.1111/jphp.12273 24835111

[B68] Huang X. Wu P. Huang F. Xu M. Chen M. Huang K. (2017). Baicalin Attenuates Chronic Hypoxia-Induced Pulmonary Hypertension via Adenosine A2a Receptor-Induced Sdf-1/cxcr4/pi3k/akt Signaling. J. Biomed. Sci. 24 (1), 52. 10.1186/s12929-017-0359-3 28774332 PMC5543745

[B69] Huang X. Zou L. Yu X. Chen M. Guo R. Cai H. (2015). Salidroside Attenuates Chronic Hypoxia-Induced Pulmonary Hypertension via Adenosine A2a Receptor Related Mitochondria-dependent Apoptosis Pathway. J. Mol. Cel Cardiol 82, 153–166. 10.1016/j.yjmcc.2015.03.005 25772255

[B70] Huang Y. Li L. Li X. Fan S. Zhuang P. Zhang Y. (2018). Ginseng Compatibility Environment Attenuates Toxicity and Keeps Efficacy in Cor Pulmonale Treated by Fuzi Beimu Incompatibility through the Coordinated Crosstalk of Pka and Epac Signaling Pathways. Front. Pharmacol. 9, 634. 10.3389/fphar.2018.00634 29962951 PMC6013823

[B71] Huang Y. F. Liu M. L. Dong M. Q. Yang W. C. Zhang B. Luan L. L. (2009). Effects of Sodium Tanshinone Ii a Sulphonate on Hypoxic Pulmonary Hypertension in Rats *In Vivo* and on kv2.1 Expression in Pulmonary Artery Smooth Muscle Cells *In Vitro* . J. Ethnopharmacol 125 (3), 436–443. 10.1016/j.jep.2009.07.020 19635545

[B72] Humbert M. Guignabert C. Bonnet S. Dorfmüller P. Klinger J. R. Nicolls M. R. (2019). Pathology and Pathobiology of Pulmonary Hypertension: State of the Art and Research Perspectives. Eur. Respir. J. 53 (1), 1801887. 10.1183/13993003.01887-2018 30545970 PMC6351340

[B73] Jiang Q. Lu W. Yang K. Hadadi C. Fu X. Chen Y. (2016). Sodium Tanshinone IIA Sulonate Inhibits Hypoxia-Induced Enhancement of SOCE in Pulmonary Arterial Smooth Muscle Cells via the PKG-PPAR-γ Signaling axis. Am. J. Physiol. Cel Physiol 311 (1), C136–C149. 10.1152/ajpcell.00252.2015 PMC4967135 27194472

[B74] Jiang Q. S. Huang X. N. Dai Z. K. Yang G. Z. Zhou Q. X. Shi J. S. (2007). Inhibitory Effect of Ginsenoside Rb1 on Cardiac Hypertrophy Induced by Monocrotaline in Rat. J. Ethnopharmacol 111 (3), 567–572. 10.1016/j.jep.2007.01.006 17374466

[B75] Jiang W. Y. (2005). Therapeutic Wisdom in Traditional Chinese Medicine: A Perspective from Modern Science. Discov. Med. 5 (11), 455–461. 10.1016/j.tips.2005.09.006 20704842

[B76] Jin H. Jiang Y. Du F. Guo L. Wang G. Kim S. C. (2019). Isoliquiritigenin Attenuates Monocrotaline-Induced Pulmonary Hypertension via Inhibition of the Inflammatory Response and Pasmcs Proliferation. Evid. Based Complement. Alternat Med. 2019, 4568198. 10.1155/2019/4568198 31239860 PMC6556334

[B77] Jin U. H. Suh S. J. Chang H. W. Son J. K. Lee S. H. Son K. H. (2008). Tanshinone Iia from Salvia Miltiorrhiza Bunge Inhibits Human Aortic Smooth Muscle Cell Migration and Mmp-9 Activity through Akt Signaling Pathway. J. Cel Biochem 104 (1), 15–26. 10.1002/jcb.21599 17979138

[B78] Kang N. Gao H. He L. Liu Y. Fan H. Xu Q. (2021). Ginsenoside Rb1 Is an Immune-Stimulatory Agent with Antiviral Activity against Enterovirus 71. J. Ethnopharmacol 266, 113401. 10.1016/j.jep.2020.113401 32980486

[B79] Kassa B. Mickael C. Kumar R. Sanders L. Koyanagi D. Hernandez-Saavedra D. (2019). Paclitaxel Blocks Th2-Mediated TGF-β Activation in Schistosoma Mansoni-Induced Pulmonary Hypertension. Pulm. Circ. 9 (1), 2045894018820813. 10.1177/2045894018820813 30511588 PMC6304706

[B80] Kaur G. Singh N. Samuel S. S. Bora H. K. Sharma S. Pachauri S. D. (2015). Withania Somnifera Shows a Protective Effect in Monocrotaline-Induced Pulmonary Hypertension. Pharm. Biol. 53 (1), 147–157. 10.3109/13880209.2014.912240 25237891

[B81] Kay J. M. Keane P. M. Suyama K. L. Gauthier D. (1982). Angiotensin Converting Enzyme Activity and Evolution of Pulmonary Vascular Disease in Rats with Monocrotaline Pulmonary Hypertension. Thorax 37 (2), 88–96. 10.1136/thx.37.2.88 6281933 PMC459256

[B82] Khan H. Saeedi M. Nabavi S. M. Mubarak M. S. Bishayee A. (2019). Glycosides from Medicinal Plants as Potential Anticancer Agents: Emerging Trends towards Future Drugs. Curr. Med. Chem. 26 (13), 2389–2406. 10.2174/0929867325666180403145137 29611474

[B83] Kim H. S. Zhang Y. H. Oh K. W. Ahn H. Y. (1997). Vasodilating and Hypotensive Effects of Fangchinoline and Tetrandrine on the Rat Aorta and the Stroke-Prone Spontaneously Hypertensive Rat. J. Ethnopharmacol 58 (2), 117–123. 10.1016/s0378-8741(97)00092-5 9406900

[B84] Kosanovic D. Tian X. Pak O. Lai Y. J. Hsieh Y. L. Seimetz M. (2013). Rhodiola: An Ordinary Plant or a Promising Future Therapy for Pulmonary Hypertension? A Brief Review. Pulm. Circ. 3 (3), 499–506. 10.1086/674303 24618536 PMC4070792

[B85] Kumar N. Goel N. (2019). Phenolic Acids: Natural Versatile Molecules with Promising Therapeutic Applications. Biotechnol. Rep. (Amst) 24, e00370. 10.1016/j.btre.2019.e00370 31516850 PMC6734135

[B86] Kuriyama S. Morio Y. Toba M. Nagaoka T. Takahashi F. Iwakami S. (2014). Genistein Attenuates Hypoxic Pulmonary Hypertension via Enhanced Nitric Oxide Signaling and the Erythropoietin System. Am. J. Physiol. Lung Cel Mol Physiol 306 (11), L996–L1005. 10.1152/ajplung.00276.2013 24705719

[B87] Kurosawa R. Satoh K. Nakata T. Shindo T. Kikuchi N. Satoh T. (2021). Identification of Celastrol as a Novel Therapeutic Agent for Pulmonary Arterial Hypertension and Right Ventricular Failure through Suppression of Bsg (Basigin)/cypa (Cyclophilin a). Arterioscler Thromb. Vasc. Biol. 41 (3), 1205–1217. 10.1161/ATVBAHA.120.315731 33472404

[B88] Kwan C. Y. Wang Z. L. (1993). Tetrandrine: A Vasodilator of Medicinal Herb Origin with a Novel Contractile Effect on Dog Saphenous Vein. Eur. J. Pharmacol. 238 (2-3), 431–434. 10.1016/0014-2999(93)90881-h 8405114

[B89] Lan T. H. Chen X. L. Wu Y. S. Qiu H. L. Li J. Z. Ruan X. M. (2018). 3,7-bis(2-hydroxyethyl)icaritin, a Potent Inhibitor of Phosphodiesterase-5, Prevents Monocrotaline-Induced Pulmonary Arterial Hypertension via No/cgmp Activation in Rats. Eur. J. Pharmacol. 829, 102–111. 10.1016/j.ejphar.2018.04.011 29665366

[B90] Lan X. Zhao J. Zhang Y. Chen Y. Liu Y. Xu F. (2020). Oxymatrine Exerts Organ- and Tissue-Protective Effects by Regulating Inflammation, Oxidative Stress, Apoptosis, and Fibrosis: From Bench to Bedside. Pharmacol. Res. 151, 104541. 10.1016/j.phrs.2019.104541 31733326

[B91] Lee D. Y. W. Li Q. Y. Liu J. Efferth T. (2021). Traditional Chinese Herbal Medicine at the Forefront Battle against Covid-19: Clinical Experience and Scientific Basis. Phytomedicine 80, 153337. 10.1016/j.phymed.2020.153337 33221457 PMC7521884

[B92] Lee F. Y. Lu H. I. Zhen Y. Y. Leu S. Chen Y. L. Tsai T. H. (2013). Benefit of Combined Therapy with Nicorandil and Colchicine in Preventing Monocrotaline-Induced Rat Pulmonary Arterial Hypertension. Eur. J. Pharm. Sci. 50 (3-4), 372–384. 10.1016/j.ejps.2013.08.004 23954457

[B93] Leopardi M. Houbballah R. Becquemin J. P. (2014). Effectiveness of Zilver Ptx Eluting Stent in Tasc C/d Lesions and Restenosis. J. Cardiovasc. Surg. (Torino) 55 (2), 229–234. 24670831

[B94] Li C. Peng G. Long J. Xiao P. Zeng X. Yang H. (2020a). Protective Effects of Resveratrol and Sr1001 on Hypoxia-Induced Pulmonary Hypertension in Rats. Clin. Exp. Hypertens. 42 (6), 519–526. 10.1080/10641963.2020.1714643 31973589

[B95] Li C. Y. Yang P. Jiang Y. L. Lin Z. Pu Y. W. Xie L. Q. (2020b). Ginsenoside Rb1 Attenuates Cardiomyocyte Apoptosis Induced by Myocardial Ischemia Reperfusion Injury through Mtor Signal Pathway. Biomed. Pharmacother. 125, 109913. 10.1016/j.biopha.2020.109913 32006902

[B96] Li L. Dong P. Hou C. Cao F. Sun S. He F. (2016a). Hydroxysafflor Yellow a (Hsya) Attenuates Hypoxic Pulmonary Arterial Remodelling and Reverses Right Ventricular Hypertrophy in Rats. J. Ethnopharmacol 186, 224–233. 10.1016/j.jep.2016.04.004 27063983

[B97] Li L. S. Luo Y. M. Liu J. Zhang Y. Fu X. X. Yang D. L. (2016b). Icariin Inhibits Pulmonary Hypertension Induced by Monocrotaline through Enhancement of No/cgmp Signaling Pathway in Rats. Evid. Based Complement. Alternat Med. 2016, 7915415. 10.1155/2016/7915415 27366192 PMC4904099

[B98] Li Q. Qiu Y. Mao M. Lv J. Zhang L. Li S. (2014). Antioxidant Mechanism of Rutin on Hypoxia-Induced Pulmonary Arterial Cell Proliferation. Molecules 19 (11), 19036–19049. 10.3390/molecules191119036 25412048 PMC6270752

[B99] Li Q. Y. Zhu Y. F. Zhang M. Chen L. Zhang Z. Du Y. L. (2015a). Chlorogenic Acid Inhibits Hypoxia-Induced Pulmonary Artery Smooth Muscle Cells Proliferation via C-Src and Shc/grb2/erk2 Signaling Pathway. Eur. J. Pharmacol. 751, 81–88. 10.1016/j.ejphar.2015.01.046 25666384

[B100] Li T. Luo X. J. Wang E. L. Li N. S. Zhang X. J. Song F. L. (2019a). Magnesium Lithospermate B Prevents Phenotypic Transformation of Pulmonary Arteries in Rats with Hypoxic Pulmonary Hypertension through Suppression of Nadph Oxidase. Eur. J. Pharmacol. 847, 32–41. 10.1016/j.ejphar.2019.01.020 30659826

[B101] Li T. Peng J. J. Wang E. L. Li N. S. Song F. L. Yang J. F. (2019b). Magnesium Lithospermate B Derived from Salvia Miltiorrhiza Ameliorates Right Ventricle Remodeling in Pulmonary Hypertensive Rats via Inhibition of Nox/vpo1 Pathway. Planta Med. 85 (9-10), 708–718. 10.1055/a-0863-4741 30822814

[B102] Li X. W. Wang X. M. Li S. Yang J. R. (2015b). Effects of Chrysin (5,7-dihydroxyflavone) on Vascular Remodeling in Hypoxia-Induced Pulmonary Hypertension in Rats. Chin. Med. 10, 4. 10.1186/s13020-015-0032-2 25722740 PMC4341233

[B103] Li Y. Song K. Zhang H. Yuan M. An N. Wei Y. (2020c). Anti-inflammatory and Immunomodulatory Effects of Baicalin in Cerebrovascular and Neurological Disorders. Brain Res. Bull. 164, 314–324. 10.1016/j.brainresbull.2020.08.016 32858128

[B104] Li Y. Wang Y. Li Y. Qian Z. Zhu L. Yang D. (2017). Osthole Attenuates Pulmonary Arterial Hypertension in Monocrotaline-treated R-ats. Mol. Med. Rep. 16 (3), 2823–2829. 10.3892/mmr.2017.6876 28677726

[B105] Lin Y. Yao J. Wu M. Ying X. Ding M. Wei Y. (2019). Tetrandrine Ameliorates Airway Remodeling of Chronic Asthma by Interfering TGF-β1/Nrf-2/ho-1 Signaling Pathway-Mediated Oxidative Stress. Can. Respir. J. 2019, 7930396. 10.1155/2019/7930396 31781316 PMC6875008

[B106] Liu C. Yang S. Wang K. Bao X. Liu Y. Zhou S. (2019a). Alkaloids from Traditional Chinese Medicine against Hepatocellular Carcinoma. Biomed. Pharmacother. 120, 109543. 10.1016/j.biopha.2019.109543 31655311

[B107] Liu C. S. Zheng Y. R. Zhang Y. F. Long X. Y. (2016). Research Progress on Berberine with a Special Focus on its Oral Bioavailability. Fitoterapia 109, 274–282. 10.1016/j.fitote.2016.02.001 26851175

[B108] Liu G. Zhang Q. Zhang J. Zhang N. (2020a). Preventive but Nontherapeutic Effect of Danshensu on Hypoxic Pulmonary Hypertension. J. Int. Med. Res. 48 (5), 300060520914218. 10.1177/0300060520914218 32419546 PMC7235679

[B109] Liu P. Yan S. Chen M. Chen A. Yao D. Xu X. (2015). Effects of Baicalin on Collagen Ι and Collagen ΙΙΙ Expression in Pulmonary Arteries of Rats with Hypoxic Pulmonary Hypertension. Int. J. Mol. Med. 35 (4), 901–908. 10.3892/ijmm.2015.2110 25716558 PMC4356435

[B110] Liu W. Fan T. Li M. Zhang G. Guo W. Yang X. (2020b). Andrographolide Potentiates Pd-1 Blockade Immunotherapy by Inhibiting Cox2-Mediated Pge2 Release. Int. Immunopharmacol 81, 106206. 10.1016/j.intimp.2020.106206 32018066

[B111] Liu X. Zhang S. Xu C. Sun Y. Sui S. Zhang Z. (2020c). The Protective of Baicalin on Myocardial Ischemia-Reperfusion Injury. Curr. Pharm. Biotechnol. 21 (13), 1386–1393. 10.2174/1389201021666200605104540 32503406

[B112] Liu Y. L. Zhou X. Y. Xuan L. J. (2019b). Magnesium Lithospermate B Ameliorates Microcirculation Perfusion in Rats by Promoting Vascular No Production via Activating the Pi3k/akt Pathway. Acta Pharmacol. Sin 40 (8), 1010–1018. 10.1038/s41401-018-0203-7 30760835 PMC6786332

[B113] Liu Y. Y. Zhang W. Y. Wang C. G. Huang J. A. Jiang J. H. Zeng D. X. (2020d). Resveratrol Prevented Experimental Pulmonary Vascular Remodeling via Mir-638 Regulating Nr4a3/cyclin D1 Pathway. Microvasc. Res. 130, 103988. 10.1016/j.mvr.2020.103988 32057731

[B114] Lu H. Zhang L. Xiao J. Wu C. Zhang H. Chen Y. (2019). Effect of Feeding Chinese Herb Medicine Ageratum-Liquid on Intestinal Bacterial Translocations Induced by H9n2 Aiv in Mice. Virol. J. 16 (1), 24. 10.1186/s12985-019-1131-y 30791956 PMC6385471

[B115] Lu Y. Wu J. Sun Y. Xin L. Jiang Z. Lin H. (2020). Qiliqiangxin Prevents Right Ventricular Remodeling by Inhibiting Apoptosis and Improving Metabolism Reprogramming with Pulmonary Arterial Hypertension. Am. J. Transl Res. 12 (9), 5655–5669. 33042446 PMC7540132

[B116] Luan F. He X. Zeng N. (2020). Tetrandrine: A Review of its Anticancer Potentials, Clinical Settings, Pharmacokinetics and Drug Delivery Systems. J. Pharm. Pharmacol. 72 (11), 1491–1512. 10.1111/jphp.13339 32696989

[B117] Luan Y. Chao S. Ju Z. Y. Wang J. Xue X. Qi T. G. (2015). Therapeutic Effects of Baicalin on Monocrotaline-Induced Pulmonary Arterial Hypertension by Inhibiting Inflammatory Response. Int. Immunopharmacol 26 (1), 188–193. 10.1016/j.intimp.2015.01.009 25601497

[B118] Luo J. Gu Y. Liu P. Jiang X. Yu W. Ye P. (2018). Berberine Attenuates Pulmonary Arterial Hypertension via Protein Phosphatase 2a Signaling Pathway Both *In Vivo* and *In Vitro* . J. Cel Physiol 233 (12), 9750–9762. 10.1002/jcp.26940 30078229

[B119] Luo Y. Xu D. Q. Dong H. Y. Zhang B. Liu Y. Niu W. (2013). Tanshinone Iia Inhibits Hypoxia-Induced Pulmonary Artery Smooth Muscle Cell Proliferation via Akt/skp2/p27-Associated Pathway. PLoS One 8 (2), e56774. 10.1371/journal.pone.0056774 23437233 PMC3578942

[B120] Matori H. Umar S. Nadadur R. D. Sharma S. Partow-Navid R. Afkhami M. (2012). Genistein, a Soy Phytoestrogen, Reverses Severe Pulmonary Hypertension and Prevents Right Heart Failure in Rats. Hypertension 60 (2), 425–430. 10.1161/HYPERTENSIONAHA.112.191445 22753213 PMC4252152

[B121] Meghwani H. Prabhakar P. Mohammed S. A. Dua P. Seth S. Hote M. P. (2018). Beneficial Effect of Ocimum Sanctum (linn) against Monocrotaline-Induced Pulmonary Hypertension in Rats. Medicines (Basel) 5 (2). 10.3390/medicines5020034 PMC6023537 29673152

[B122] Meghwani H. Prabhakar P. Mohammed S. A. Seth S. Hote M. P. Banerjee S. K. (2017). Beneficial Effects of Aqueous Extract of Stem Bark of terminalia Arjuna (roxb.), an Ayurvedic Drug in Experimental Pulmonary Hypertension. J. Ethnopharmacol 197, 184–194. 10.1016/j.jep.2016.07.029 27401289

[B123] Miao Q. Shi X. P. Ye M. X. Zhang J. Miao S. Wang S. W. (2012). Polydatin Attenuates Hypoxic Pulmonary Hypertension and Reverses Remodeling through Protein Kinase C Mechanisms. Int. J. Mol. Sci. 13 (6), 7776–7787. 10.3390/ijms13067776 22837726 PMC3397558

[B124] Mondal A. Gandhi A. Fimognari C. Atanasov A. G. Bishayee A. (2019). Alkaloids for Cancer Prevention and Therapy: Current Progress and Future Perspectives. Eur. J. Pharmacol. 858, 172472. 10.1016/j.ejphar.2019.172472 31228447

[B125] Nan X. Su S. Ma K. Ma X. Wang X. Zhaxi D. (2018). Bioactive Fraction of Rhodiola Algida against Chronic Hypoxia-Induced Pulmonary Arterial Hypertension and its Anti-proliferation Mechanism in Rats. J. Ethnopharmacol 216, 175–183. 10.1016/j.jep.2018.01.010 29325918

[B126] Nie X. Dai Y. Tan J. Chen Y. Qin G. Mao W. (2017). α-Solanine Reverses Pulmonary Vascular Remodeling and Vascular Angiogenesis in Experimental Pulmonary Artery Hypertension. J. Hypertens. 35 (12), 2419–2435. 10.1097/HJH.0000000000001475 28704260

[B127] Nik Salleh N. N. H. Othman F. A. Kamarudin N. A. Tan S. C. (2020). The Biological Activities and Therapeutic Potentials of Baicalein Extracted from Oroxylum Indicum: A Systematic Review. Molecules 25 (23). 10.3390/molecules25235677 PMC7730069 33276419

[B128] Pacheco-Ordaz R. Wall-Medrano A. Goñi M. G. Ramos-Clamont-Montfort G. Ayala-Zavala J. F. González-Aguilar G. A. (2018). Effect of Phenolic Compounds on the Growth of Selected Probiotic and Pathogenic Bacteria. Lett. Appl. Microbiol. 66 (1), 25–31. 10.1111/lam.12814 29063625

[B129] Paffett M. L. Lucas S. N. Campen M. J. (2012). Resveratrol Reverses Monocrotaline-Induced Pulmonary Vascular and Cardiac Dysfunction: A Potential Role for Atrogin-1 in Smooth Muscle. Vascul Pharmacol. 56 (1-2), 64–73. 10.1016/j.vph.2011.11.002 22146233 PMC3370653

[B130] Qin N. Gong Q. H. Wei L. W. Wu Q. Huang X. N. (2008). Total Ginsenosides Inhibit the Right Ventricular Hypertrophy Induced by Monocrotaline in Rats. Biol. Pharm. Bull. 31 (8), 1530–1535. 10.1248/bpb.31.1530 18670084

[B131] Qin N. Lu X. Liu Y. Qiao Y. Qu W. Feng F. (2021). Recent Research Progress of Uncaria Spp. Based on Alkaloids: Phytochemistry, Pharmacology and Structural Chemistry. Eur. J. Med. Chem. 210, 112960. 10.1016/j.ejmech.2020.112960 33148492

[B132] Qin N. Yang W. Feng D. Wang X. Qi M. Du T. (2016). Total Ginsenosides Suppress Monocrotaline-Induced Pulmonary Hypertension in Rats: Involvement of Nitric Oxide and Mitogen-Activated Protein Kinase Pathways. J. Ginseng Res. 40 (3), 285–291. 10.1016/j.jgr.2015.09.005 27616905 PMC5005363

[B133] Qu C. Xu Y. Yang X. Lu X. (2020). Magnesium Lithospermate B Improves Pulmonary Artery Banding Induced Right Ventricular Dysfunction by Alleviating Inflammation via P38mapk Pathway. Pulm. Pharmacol. Ther. 63, 101935. 10.1016/j.pupt.2020.101935 32783991

[B134] Rajabi S. Najafipour H. Jafarinejad Farsangi S. Joukar S. Beik A. Iranpour M. (2020). Perillyle Alcohol and Quercetin Ameliorate Monocrotaline-Induced Pulmonary Artery Hypertension in Rats through Parp1-Mediated Mir-204 Down-Regulation and its Downstream Pathway. BMC Complement. Med. Ther. 20 (1), 218. 10.1186/s12906-020-03015-1 32660602 PMC7359282

[B135] Rajabi S. Najafipour H. Jafarinejad-Farsangi S. Joukar S. Beik A. Askaripour M. (2021). Quercetin, Perillyl Alcohol, and Berberine Ameliorate Right Ventricular Disorders in Experimental Pulmonary Arterial Hypertension: Effects on Mir-204, Mir-27a, Fibrotic, Apoptotic, and Inflammatory Factors. J. Cardiovasc. Pharmacol. 77 (6), 777–786. 10.1097/fjc.0000000000001015 34016844

[B136] Rakotomalala G. Agard C. Tonnerre P. Tesse A. Derbré S. Michalet S. (2013). Extract from Mimosa Pigra Attenuates Chronic Experimental Pulmonary Hypertension. J. Ethnopharmacol 148 (1), 106–116. 10.1016/j.jep.2013.03.075 23583901

[B137] Rice K. M. Manne N. D. Kolli M. B. Wehner P. S. Dornon L. Arvapalli R. (2016). Curcumin Nanoparticles Attenuate Cardiac Remodeling Due to Pulmonary Arterial Hypertension. Artif. Cell Nanomed Biotechnol 44 (8), 1909–1916. 10.3109/21691401.2015.1111235 26631548

[B138] Rosenkranz S. Howard L. S. Gomberg-Maitland M. Hoeper M. M. (2020). Systemic Consequences of Pulmonary Hypertension and Right-Sided Heart Failure. Circulation 141 (8), 678–693. 10.1161/circulationaha.116.022362 32091921

[B139] Shao J. Wang P. Liu A. Du X. Bai J. Chen M. (2016). Punicalagin Prevents Hypoxic Pulmonary Hypertension via Anti-oxidant Effects in Rats. Am. J. Chin. Med. 44 (4), 785–801. 10.1142/S0192415X16500439 27222062

[B140] Shi R. Wei Z. Zhu D. Fu N. Wang C. Yin S. (2018a). Baicalein Attenuates Monocrotaline-Induced Pulmonary Arterial Hypertension by Inhibiting Vascular Remodeling in Rats. Pulm. Pharmacol. Ther. 48, 124–135. 10.1016/j.pupt.2017.11.003 29133079

[B141] Shi R. Zhu D. Wei Z. Fu N. Wang C. Liu L. (2018b). Baicalein Attenuates Monocrotaline-Induced Pulmonary Arterial Hypertension by Inhibiting Endothelial-To-Mesenchymal Transition. Life Sci. 207, 442–450. 10.1016/j.lfs.2018.06.033 29969608

[B142] Singh S. Meena A. Luqman S. (2021). Baicalin Mediated Regulation of Key Signaling Pathways in Cancer. Pharmacol. Res. 164, 105387. 10.1016/j.phrs.2020.105387 33352232

[B143] Sun Y. Lu M. Sun T. Wang H. (2021). Astragaloside Iv Attenuates Inflammatory Response Mediated by Nlrp-3/calpain-1 Is Involved in the Development of Pulmonary Hypertension. J. Cel Mol Med 25 (1), 586–590. 10.1111/jcmm.15671 PMC7810938 33295020

[B144] Tang B. Chen G. X. Liang M. Y. Yao J. P. Wu Z. K. (2015). Ellagic Acid Prevents Monocrotaline-Induced Pulmonary Artery Hypertension via Inhibiting Nlrp3 Inflammasome Activation in Rats. Int. J. Cardiol. 180, 134–141. 10.1016/j.ijcard.2014.11.161 25438234

[B145] Tang J. L. Liu B. Y. Ma K. W. (2008). Traditional Chinese Medicine. Lancet 372 (9654), 1938–1940. 10.1016/s0140-6736(08)61354-9 18930523

[B146] Tang M. Wang R. Feng P. Dong Q. Chen W. Zhao Y. (2020). Dihydroartemisinin Attenuates Pulmonary Hypertension through Inhibition of Pulmonary Vascular Remodeling in Rats. J. Cardiovasc. Pharmacol. 76 (3), 337–348. 10.1097/fjc.0000000000000862 32569012

[B147] Tello K. Seeger W. Naeije R. Vanderpool R. Ghofrani H. A. Richter M. (2021). Right Heart Failure in Pulmonary Hypertension: Diagnosis and New Perspectives on Vascular and Direct Right Ventricular Treatment. Br. J. Pharmacol. 178 (1), 90–107. 10.1111/bph.14866 31517994

[B148] Thangavel P. Puga-Olguín A. Rodríguez-Landa J. F. Zepeda R. C. (2019). Genistein as Potential Therapeutic Candidate for Menopausal Symptoms and Other Related Diseases. Molecules 24 (21), 3892. 10.3390/molecules24213892 PMC6864469 31671813

[B149] Thenappan T. Khoruts A. Chen Y. Weir E. K. (2019). Can Intestinal Microbiota and Circulating Microbial Products Contribute to Pulmonary Arterial Hypertension? Am. J. Physiol. Heart Circ. Physiol. 317 (5), H1093–H1101. 10.1152/ajpheart.00416.2019 31490732 PMC6879928

[B150] Tsai H. H. Chen I. J. Lo Y. C. (2008). Effects of San-Huang-Xie-Xin-Tang on U46619-Induced Increase in Pulmonary Arterial Blood Pressure. J. Ethnopharmacol 117 (3), 457–462. 10.1016/j.jep.2008.02.024 18387761

[B151] Tu Y. (2011). The Discovery of Artemisinin (Qinghaosu) and Gifts from Chinese Medicine. Nat. Med. 17 (10), 1217–1220. 10.1038/nm.2471 21989013

[B152] Tuli H. S. Aggarwal V. Kaur J. Aggarwal D. Parashar G. Parashar N. C. (2020). Baicalein: A Metabolite with Promising Antineoplastic Activity. Life Sci. 259, 118183. 10.1016/j.lfs.2020.118183 32781058

[B153] Vázquez-Garza E. Bernal-Ramírez J. Jerjes-Sánchez C. Lozano O. Acuña-Morín E. Vanoye-Tamez M. (2020). Resveratrol Prevents Right Ventricle Remodeling and Dysfunction in Monocrotaline-Induced Pulmonary Arterial Hypertension with a Limited Improvement in the Lung Vasculature. Oxid Med. Cel Longev 2020, 1841527. 10.1155/2020/1841527 PMC7023844 32089765

[B154] Wande Y. Jie L. Aikai Z. Yaguo Z. Linlin Z. Yue G. (2020). Berberine Alleviates Pulmonary Hypertension through Trx1 and β-catenin Signaling Pathways in Pulmonary Artery Smooth Muscle Cells. Exp. Cel Res 390 (1), 111910. 10.1016/j.yexcr.2020.111910 32147507

[B155] Wang H. L. Zhang X. H. Chang T. H. (2002). Effects of Tetrandrine on Smooth Muscle Contraction Induced by Mediators in Pulmonary Hypertension. Acta Pharmacol. Sin 23 (12), 1114–1120. 12466049

[B156] Wang J. Dong M. Q. Liu M. L. Xu D. Q. Luo Y. Zhang B. (2010). Tanshinone Iia Modulates Pulmonary Vascular Response to Agonist and Hypoxia Primarily via Inhibiting Ca2+ Influx and Release in normal and Hypoxic Pulmonary Hypertension Rats. Eur. J. Pharmacol. 640 (1-3), 129–138. 10.1016/j.ejphar.2010.04.047 20460121

[B157] Wang J. Jiang Q. Wan L. Yang K. Zhang Y. Chen Y. (2013). Sodium Tanshinone Iia Sulfonate Inhibits Canonical Transient Receptor Potential Expression in Pulmonary Arterial Smooth Muscle from Pulmonary Hypertensive Rats. Am. J. Respir. Cel Mol Biol 48 (1), 125–134. 10.1165/rcmb.2012-0071OC PMC3547081 23065131

[B158] Wang J. Wong Y. K. Liao F. (2018a). What Has Traditional Chinese Medicine Delivered for Modern Medicine? Expert Rev. Mol. Med. 20, e4. 10.1017/erm.2018.3 29747718

[B159] Wang J. Xu J. Gong X. Yang M. Zhang C. Li M. (2019a). Biosynthesis, Chemistry, and Pharmacology of Polyphenols from Chinese Salvia Species: A Review. Molecules 24 (1), 155. 10.3390/molecules24010155 PMC6337547 30609767

[B160] Wang M. Yang L. Yang J. Zhou Y. Wang C. (2019b). Magnesium Lithospermate B Attenuates Renal Injury in 5/6 Renal Ablation/infarction Rats by Mitochondrial Pathway of Apoptosis. Biomed. Pharmacother. 118, 109316. 10.1016/j.biopha.2019.109316 31387002

[B161] Wang R. X. He R. L. Jiao H. X. Dai M. Mu Y. P. Hu Y. (2015a). Ginsenoside Rb1 Attenuates Agonist-Induced Contractile Response via Inhibition of Store-Operated Calcium Entry in Pulmonary Arteries of normal and Pulmonary Hypertensive Rats. Cell Physiol Biochem 35 (4), 1467–1481. 10.1159/000373966 25791507

[B162] Wang S. Zhang S. Wang S. Gao P. Dai L. (2020a). A Comprehensive Review on Pueraria: Insights on its Chemistry and Medicinal Value. Biomed. Pharmacother. 131, 110734. 10.1016/j.biopha.2020.110734 32942158

[B163] Wang T. Hou J. Xiao W. Zhang Y. Zhou L. Yuan L. (2020b). Chinese Medicinal Plants for the Potential Management of High-Altitude Pulmonary Oedema and Pulmonary Hypertension. Pharm. Biol. 58 (1), 815–827. 10.1080/13880209.2020.1804407 32883127 PMC8641673

[B164] Wang X. Cai X. Wang W. Jin Y. Chen M. Huang X. (2018b). Effect of Asiaticoside on Endothelial Cells in Hypoxia-induced P-ulmonary H-ypertension. Mol. Med. Rep. 17 (2), 2893–2900. 10.3892/mmr.2017.8254 29257311 PMC5783505

[B165] Wang X. Xiao D. Ma C. Zhang L. Duan Q. Zheng X. (2019c). The Effect of Honokiol on Pulmonary Artery Endothelium Cell Autophagy Mediated by Cyclophilin a in Hypoxic Pulmonary Arterial Hypertension. J. Pharmacol. Sci. 139 (3), 158–165. 10.1016/j.jphs.2019.01.005 30770282

[B166] Wang X. Yang Y. Yang D. Tong G. Lv S. Lin X. (2016a). Tetrandrine Prevents Monocrotaline-Induced Pulmonary Arterial Hypertension in Rats through Regulation of the Protein Expression of Inducible Nitric Oxide Synthase and Cyclic Guanosine Monophosphate-dependent Protein Kinase Type 1. J. Vasc. Surg. 64 (5), 1468–1477. 10.1016/j.jvs.2015.09.016 26527422

[B167] Wang X. B. Wang W. Zhu X. C. Ye W. J. Cai H. Wu P. L. (2015b). The Potential of Asiaticoside for TGF-β1/Smad Signaling Inhibition in Prevention and Progression of Hypoxia-Induced Pulmonary Hypertension. Life Sci. 137, 56–64. 10.1016/j.lfs.2015.07.016 26209140

[B168] Wang Y. Cao S. H. Cui Y. J. Kong L. K. Tian H. Cai H. X. (2015c). Salvia Miltiorrhiza bge.F.Alba Ameliorates the Progression of Monocrotaline-Induced Pulmonary Hypertension by Protecting Endothelial Injury in Rats. Tohoku J. Exp. Med. 236 (2), 155–162. 10.1620/tjem.236.155 26074502

[B169] Wang Y. Ma T. T. Gao N. N. Zhou X. L. Jiang H. Guo R. (2016b). Effect of Tongxinluo on Pulmonary Hypertension and Pulmonary Vascular Remodeling in Rats Exposed to a Low Pressure Hypoxic Environment. J. Ethnopharmacol 194, 668–673. 10.1016/j.jep.2016.10.004 27737815

[B170] Warowicka A. Nawrot R. Goździcka-Józefiak A. (2020). Antiviral Activity of Berberine. Arch. Virol. 165 (9), 1935–1945. 10.1007/s00705-020-04706-3 32594322 PMC7320912

[B171] Wei Y. Wu Y. Feng K. Zhao Y. Tao R. Xu H. (2020). Astragaloside IV Inhibits Cardiac Fibrosis via miR-135a-TRPM7-TGF-β/Smads Pathway. J. Ethnopharmacol 249, 112404. 10.1016/j.jep.2019.112404 31739105

[B172] Wilson D. N. Schacht S. E. Al-Nakkash L. Babu J. R. Broderick T. L. (2016). Resveratrol Prevents Pulmonary Trunk Remodeling but Not Right Ventricular Hypertrophy in Monocrotaline-Induced Pulmonary Hypertension. Pathophysiology 23 (4), 243–250. 10.1016/j.pathophys.2016.05.004 27374951

[B173] Wisutthathum S. Demougeot C. Totoson P. Adthapanyawanich K. Ingkaninan K. Temkitthawon P. (2018). Eulophia Macrobulbon Extract Relaxes Rat Isolated Pulmonary Artery and Protects against Monocrotaline-Induced Pulmonary Arterial Hypertension. Phytomedicine 50, 157–165. 10.1016/j.phymed.2018.05.014 30466974

[B174] Wu F. Yao W. Yang J. Zhang M. Xu Y. Hao Y. (2017a). Protective Effects of Aloperin on Monocroline-Induced Pulmonary Hypertension via Regulation of Rho A/rho Kinsase Pathway in Rats. Biomed. Pharmacother. 95, 1161–1168. 10.1016/j.biopha.2017.08.126 28926926

[B175] Wu J. Jia J. Liu L. Yang F. Fan Y. Zhang S. (2017b). Schisandrin B Displays a Protective Role against Primary Pulmonary Hypertension by Targeting Transforming Growth Factor β1. J. Am. Soc. Hypertens. 11 (3), 148–e1. 10.1016/j.jash.2016.12.007 28117274

[B176] Wu P. Xie X. Chen M. Sun J. Cai L. Wei J. (2021). Elucidation of the Mechanisms and Molecular Targets of Qishen Yiqi Formula for the Treatment of Pulmonary Arterial Hypertension Using a Bioinformatics/network Topology-Based Strategy. Comb. Chem. High Throughput Screen. 24 (5), 701–715. 10.2174/1386207323666201019145354 33076804

[B177] Wu Y. Xu S. Tian X. Y. (2020). The Effect of Salvianolic Acid on Vascular protection and Possible Mechanisms. Oxid Med. Cel Longev 2020, 5472096. 10.1155/2020/5472096 PMC7533016 33062143

[B178] Xiang L. Li Y. Deng X. Kosanovic D. Schermuly R. T. Li X. (2018). Natural Plant Products in Treatment of Pulmonary Arterial Hypertension. Pulm. Circ. 8 (3), 2045894018784033. 10.1177/2045894018784033 29869936 PMC6055327

[B179] Xiang Y. Cai C. Wu Y. Yang L. Ye S. Zhao H. (2020). Icariin Attenuates Monocrotaline-Induced Pulmonary Arterial Hypertension via the Inhibition of TGF-β1/Smads Pathway in Rats. Evid. Based Complement. Alternat Med. 2020, 9238428. 10.1155/2020/9238428 33335559 PMC7723481

[B180] Xin L. Gao J. Lin H. Qu Y. Shang C. Wang Y. (2020). Regulatory Mechanisms of Baicalin in Cardiovascular Diseases: A Review. Front. Pharmacol. 11, 583200. 10.3389/fphar.2020.583200 33224035 PMC7667240

[B181] Xu C. Hou B. He P. Ma P. Yang X. Yang X. (2020). Neuroprotective Effect of Salvianolic Acid a against Diabetic Peripheral Neuropathy through Modulation of Nrf2. Oxid Med. Cel Longev 2020, 6431459. 10.1155/2020/6431459 PMC7063195 32184918

[B182] Xu D. Li Y. Zhang B. Wang Y. Liu Y. Luo Y. (2016). Resveratrol Alleviate Hypoxic Pulmonary Hypertension via Anti-inflammation and Anti-oxidant Pathways in Rats. Int. J. Med. Sci. 13 (12), 942–954. 10.7150/ijms.16810 27994500 PMC5165688

[B183] Xu Y. Gu Q. Qu C. (2017). Capsaicin Pretreatment Reversed Pulmonary Arterial Hypertension by Alleviating Inflammation via P38mapk Pathway. Exp. Lung Res. 43 (1), 8–18. 10.1080/01902148.2016.1271481 28281854

[B184] Xu Y. Lin L. Tang L. Zheng M. Ma Y. Huang L. (2014). Notoginsenoside R1 Attenuates Hypoxia and Hypercapnia-Induced Vasoconstriction in Isolated Rat Pulmonary Arterial Rings by Reducing the Expression of Erk. Am. J. Chin. Med. 42 (4), 799–816. 10.1142/S0192415X14500517 25004876

[B185] Xue X. Zhang S. Jiang W. Wang J. Xin Q. Sun C. (2021). Protective Effect of Baicalin against Pulmonary Arterial Hypertension Vascular Remodeling through Regulation of TNF-α Signaling Pathway. Pharmacol. Res. Perspect. 9 (1), e00703. 10.1002/prp2.703 33421306 PMC7796790

[B186] Yang D. L. Zhang H. G. Xu Y. L. Gao Y. H. Yang X. J. Hao X. Q. (2010). Resveratrol Inhibits Right Ventricular Hypertrophy Induced by Monocrotaline in Rats. Clin. Exp. Pharmacol. Physiol. 37 (2), 150–155. 10.1111/j.1440-1681.2009.05231.x 19566840

[B187] Yang J. (2017). Observation of the Effects of the Suhuang Zhike Capsule on Acute Bronchitis. J. Biol. Regul. Homeost Agents 31 (2), 453–457. 28685552

[B188] Yang J. Tian S. Zhao J. Zhang W. (2020a). Exploring the Mechanism of Tcm Formulae in the Treatment of Different Types of Coronary Heart Disease by Network Pharmacology and Machining Learning. Pharmacol. Res. 159, 105034. 10.1016/j.phrs.2020.105034 32565312

[B189] Yang J. M. Zhou R. Zhang M. Tan H. R. Yu J. Q. (2018). Betaine Attenuates Monocrotaline-Induced Pulmonary Arterial Hypertension in Rats via Inhibiting Inflammatory Response. Molecules 23 (6), 1274. 10.3390/molecules23061274 PMC6100216 29861433

[B190] Yang P. S. Kim D. H. Lee Y. J. Lee S. E. Kang W. J. Chang H. J. (2014). Glycyrrhizin, Inhibitor of High Mobility Group Box-1, Attenuates Monocrotaline-Induced Pulmonary Hypertension and Vascular Remodeling in Rats. Respir. Res. 15, 148. 10.1186/s12931-014-0148-4 25420924 PMC4248446

[B191] Yang W. Ip S. P. Liu L. Xian Y. F. Lin Z. X. (2020b). Uncaria Rhynchophylla and its Major Constituents on central Nervous System: A Review on Their Pharmacological Actions. Curr. Vasc. Pharmacol. 18 (4), 346–357. 10.2174/1570161117666190704092841 31272356

[B192] Yang Z. B. Luo X. J. Ren K. D. Peng J. J. Tan B. Liu B. (2015). Beneficial Effect of Magnesium Lithospermate B on Cerebral Ischemia-Reperfusion Injury in Rats Involves the Regulation of Mir-107/glutamate Transporter 1 Pathway. Eur. J. Pharmacol. 766, 91–98. 10.1016/j.ejphar.2015.09.042 26420356

[B193] Yao J. Fang X. Zhang C. Yang Y. Wang D. Chen Q. (2021). Astragaloside IV Attenuates Hypoxia-induced P-ulmonary V-ascular R-emodeling via the Notch S-ignaling P-athway. Mol. Med. Rep. 23 (1), 1. 10.3892/mmr.2020.11726 PMC7716412 33236156

[B194] Yao L. Yang Y. He G. Ou C. Wang L. Liu K. (2018). Global Proteomics Deciphered Novel-Function of Osthole against Pulmonary Arterial Hypertension. Sci. Rep. 8 (1), 5556. 10.1038/s41598-018-23775-8 29615702 PMC5882969

[B195] Yin F. Zhou H. Fang Y. Li C. He Y. Yu L. (2020). Astragaloside Iv Alleviates Ischemia Reperfusion-Induced Apoptosis by Inhibiting the Activation of Key Factors in Death Receptor Pathway and Mitochondrial Pathway. J. Ethnopharmacol 248, 112319. 10.1016/j.jep.2019.112319 31639488

[B196] Yu H. Liu J. Dong Y. Xu M. Xu L. Guan H. (2018). Anti-hypoxic Effect of Dihydroartemisinin on Pulmonary Artery Endothelial Cells. Biochem. Biophys. Res. Commun. 506 (4), 840–846. 10.1016/j.bbrc.2018.10.176 30391003

[B197] Yu L. Tu Y. Jia X. Fang K. Liu L. Wan L. (2017). Resveratrol Protects against Pulmonary Arterial Hypertension in Rats via Activation of Silent Information Regulator 1. Cel Physiol Biochem 42 (1), 55–67. 10.1159/000477115 28494457

[B198] Yu M. Peng L. Liu P. Yang M. Zhou H. Ding Y. (2020). Paeoniflorin ameliorates chronic hypoxia/su5416-induced pulmonary arterial hypertension by inhibiting endothelial-to-mesenchymal transition. Drug Des. Devel Ther. 14, 1191–1202. 10.2147/DDDT.S235207 PMC7090222 32256050

[B199] Yuan L. B. Hua C. Y. Gao S. Yin Y. L. Dai M. Meng H. Y. (2017a). Astragalus Polysaccharides Attenuate Monocrotaline-Induced Pulmonary Arterial Hypertension in Rats. Am. J. Chin. Med. 45 (4), 773–789. 10.1142/S0192415X17500410 28521513

[B200] Yuan T. Chen Y. Zhang H. Fang L. Du G. (2017b). Salvianolic Acid a, a Component of Salvia Miltiorrhiza, Attenuates Endothelial-Mesenchymal Transition of Hpaecs Induced by Hypoxia. Am. J. Chin. Med. 45 (6), 1185–1200. 10.1142/S0192415X17500653 28893092

[B201] Yuan T. Zhang H. Chen D. Chen Y. Lyu Y. Fang L. (2019). Puerarin Protects Pulmonary Arteries from Hypoxic Injury through the BMPRII and PPARγ Signaling Pathways in Endothelial Cells. Pharmacol. Rep. 71 (5), 855–861. 10.1016/j.pharep.2019.05.002 31408784

[B202] Yue Y. Li Y. Q. Fu S. Wu Y. T. Zhu L. Hua L. (2020). Osthole Inhibits Cell Proliferation by Regulating the TGF-β1/Smad/p38 Signaling Pathways in Pulmonary Arterial Smooth Muscle Cells. Biomed. Pharmacother. 121, 109640. 10.1016/j.biopha.2019.109640 31810114

[B203] Zhai Z. Wang J. Zhao L. Yuan J. X. Wang C. (2010). Pulmonary Hypertension in china: Pulmonary Vascular Disease: The Global Perspective. Chest 137 (6 Suppl. l), 69s–77s. 10.1378/chest.09-2802 20522582

[B204] Zhang B. Niu W. Xu D. Li Y. Liu M. Wang Y. (2014a). Oxymatrine Prevents Hypoxia- and Monocrotaline-Induced Pulmonary Hypertension in Rats. Free Radic. Biol. Med. 69, 198–207. 10.1016/j.freeradbiomed.2014.01.013 24440469

[B205] Zhang J. Dong J. Martin M. He M. Gongol B. Marin T. L. (2018a). Amp-activated Protein Kinase Phosphorylation of Angiotensin-Converting Enzyme 2 in Endothelium Mitigates Pulmonary Hypertension. Am. J. Respir. Crit. Care Med. 198 (4), 509–520. 10.1164/rccm.201712-2570OC 29570986 PMC6118028

[B206] Zhang J. Wu C. Gao L. Du G. Qin X. (2020a). Astragaloside Iv Derived from astragalus Membranaceus: A Research Review on the Pharmacological Effects. Adv. Pharmacol. 87, 89–112. 10.1016/bs.apha.2019.08.002 32089240

[B207] Zhang J. Zhang Q. Liu G. Zhang N. (2019a). Therapeutic Potentials and Mechanisms of the Chinese Traditional Medicine Danshensu. Eur. J. Pharmacol. 864, 172710. 10.1016/j.ejphar.2019.172710 31586468

[B208] Zhang L. Deng M. Zhou S. (2011). Tetramethylpyrazine Inhibits Hypoxia-Induced Pulmonary Vascular Leakage in Rats via the Ros-Hif-Vegf Pathway. Pharmacology 87 (5-6), 265–273. 10.1159/000326082 21494058

[B209] Zhang L. Ma C. Gu R. Zhang M. Wang X. Yang L. (2018b). Paeonol Regulates Hypoxia-Induced Proliferation of Pulmonary Artery Smooth Muscle Cells via Ekr 1/2 Signalling. Eur. J. Pharmacol. 834, 257–265. 10.1016/j.ejphar.2018.07.017 30053410

[B210] Zhang L. Pu Z. Wang J. Zhang Z. Hu D. Wang J. (2014b). Baicalin Inhibits Hypoxia-Induced Pulmonary Artery Smooth Muscle Cell Proliferation via the AKT/HIF-1α/p27-associated Pathway. Int. J. Mol. Sci. 15 (5), 8153–8168. 10.3390/ijms15058153 24821539 PMC4057725

[B211] Zhang M. Chang Z. Zhao F. Zhang P. Hao Y.-J. Yan L. (2019b). Protective Effects of 18β-Glycyrrhetinic Acid on Monocrotaline-Induced Pulmonary Arterial Hypertension in Rats. Front. Pharmacol. 10, 13. 10.3389/fphar.2019.00013 30723409 PMC6349717

[B212] Zhang N. Dong M. Luo Y. Zhao F. Li Y. (2018c). Danshensu Prevents Hypoxic Pulmonary Hypertension in Rats by Inhibiting the Proliferation of Pulmonary Artery Smooth Muscle Cells via TGF-β-Smad3-Associated Pathway. Eur. J. Pharmacol. 820, 1–7. 10.1016/j.ejphar.2017.12.010 29221952

[B213] Zhang Q. Fan K. Wang P. Yu J. Liu R. Qi H. (2016). Carvacrol Induces the Apoptosis of Pulmonary Artery Smooth Muscle Cells under Hypoxia. Eur. J. Pharmacol. 770, 134–146. 10.1016/j.ejphar.2015.11.037 26607464

[B214] Zhang T. Kawaguchi N. Tsuji K. Hayama E. Furutani Y. Sugiyama H. (2020b). Silibinin Upregulates Cxcr4 Expression in Cultured Bone Marrow Cells (Bmcs) Especially in Pulmonary Arterial Hypertension Rat Model. Cells 9 (5). 10.3390/cells9051276 PMC7290890 32455728

[B215] Zhang X. Chen J. Xu P. Tian X. (2018d). Protective Effects of Astragaloside Iv against Hypoxic Pulmonary Hypertension. Medchemcomm 9 (10), 1715–1721. 10.1039/c8md00341f 30429976 PMC6194492

[B216] Zhang X. Liu Q. Zhang C. Sheng J. Li S. Li W. (2019c). Puerarin Prevents Progression of Experimental Hypoxia-Induced Pulmonary Hypertension via Inhibition of Autophagy. J. Pharmacol. Sci. 141 (2), 97–105. 10.1016/j.jphs.2019.09.010 31640920

[B217] Zhang Y. Cui Y. Deng W. Wang H. Qin W. Huang C. (2017a). Isoquercitrin Protects against Pulmonary Hypertension via Inhibiting Pasmcs Proliferation. Clin. Exp. Pharmacol. Physiol. 44 (3), 362–370. 10.1111/1440-1681.12705 27873355

[B218] Zhang Y. Lu P. Qin H. Zhang Y. Sun X. Song X. (2021). Traditional Chinese Medicine Combined with Pulmonary Drug Delivery System and Idiopathic Pulmonary Fibrosis: Rationale and Therapeutic Potential. Biomed. Pharmacother. 133, 111072. 10.1016/j.biopha.2020.111072 33378971 PMC7836923

[B219] Zhang Z. Zhang L. Sun C. Kong F. Wang J. Xin Q. (2017b). Baicalin Attenuates Monocrotaline-Induced Pulmonary Hypertension through Bone Morphogenetic Protein Signaling Pathway. Oncotarget 8 (38), 63430–63441. 10.18632/oncotarget.18825 28969002 PMC5609934

[B220] Zhao F. Wang P. Jiao Y. Zhang X. Chen D. Xu H. (2020). Hydroxysafflor Yellow a: A Systematical Review on Botanical Resources, Physicochemical Properties, Drug Delivery System, Pharmacokinetics, and Pharmacological Effects. Front. Pharmacol. 11, 579332. 10.3389/fphar.2020.579332 33536906 PMC7849182

[B221] Zhao G. He F. Wu C. Li P. Li N. Deng J. (2018). Betaine in Inflammation: Mechanistic Aspects and Applications. Front. Immunol. 9, 1070. 10.3389/fimmu.2018.01070 29881379 PMC5976740

[B222] Zhao J. Yang M. Wu X. Yang Z. Jia P. Sun Y. (2019). Effects of Paclitaxel Intervention on Pulmonary Vascular Remodeling in Rats with Pulmonary Hypertension. Exp. Ther. Med. 17 (2), 1163–1170. 10.3892/etm.2018.7045 30679989 PMC6327549

[B223] Zhao S. Zheng M. X. Chen H. E. Wu C. Y. Wang W. T. (2015). Effect of Panax Notoginseng Saponins Injection on the P38mapk Pathway in Lung Tissue in a Rat Model of Hypoxic Pulmonary Hypertension. Chin. J. Integr. Med. 21 (2), 147–151. 10.1007/s11655-014-1790-2 25523598

[B224] Zheng L. Liu M. Wei M. Liu Y. Dong M. Luo Y. (2015). Tanshinone Iia Attenuates Hypoxic Pulmonary Hypertension via Modulating Kv Currents. Respir. Physiol. Neurobiol. 205, 120–128. 10.1016/j.resp.2014.09.025 25305099

[B225] Zhou A. M. Xiang Y. J. Liu E. Q. Cai C. H. Wu Y. H. Yang L. B. (2020). Salvianolic Acid a Inhibits Platelet Activation and Aggregation in Patients with Type 2 Diabetes Mellitus. BMC Cardiovasc. Disord. 20 (1), 15. 10.1186/s12872-019-01316-z 31931718 PMC6956554

[B226] Zhou Z. Y. Zhao W. R. Zhang J. Chen X. L. Tang J. Y. (2019). Sodium Tanshinone Iia Sulfonate: A Review of Pharmacological Activity and Pharmacokinetics. Biomed. Pharmacother. 118, 109362. 10.1016/j.biopha.2019.109362 31545252

[B227] Zhuang P. Huang Y. Lu Z. Yang Z. Xu L. Sun F. (2016). Camp-pka-camkii Signaling Pathway Is Involved in Aggravated Cardiotoxicity during Fuzi and Beimu Combination Treatment of Experimental Pulmonary Hypertension. Sci. Rep. 6, 34903. 10.1038/srep34903 27739450 PMC5064387

